# Reactivation of the embryonic ζ-globin gene ameliorates severe forms of α-thalassemia

**DOI:** 10.1038/s41588-026-02770-0

**Published:** 2026-10-01

**Authors:** Siyu Liu, Ayesha Ejaz, Andrew J. King, Aude-Anais Olijnik, Jacqueline Sloane-Stanley, Caroline Scott, Ali Amid, Daniel Biggs, Benjamin Davies, Jennifer Eglinton, Gabriella Martyn, Merlin Crossley, Christian Babbs, Mira T. Kassouf, Douglas R. Higgs

**Affiliations:** 1 MRC Weatherall Institute of Molecular Medicine, Radcliffe Department of Medicine, University of Oxford, Oxford, UK; 2 Chinese Academy of Medical Sciences Oxford Institute, Nuffield Department of Medicine, University of Oxford, Oxford, UK; 3 Division of Pediatric Hematology/Oncology, Department of Pediatrics, BC Children’s Hospital, University of British Columbia, Vancouver, British Columbia Canada; 4 Centre for Human Genetics, Nuffield Department of Medicine, University of Oxford, Oxford, UK; 5 Department of Haematology, John Radcliffe Hospital, Oxford, UK; 6 School of Biotechnology and Biomolecular Sciences, University of New South Wales, Sydney, New South Wales Australia

**Keywords:** Gene regulation, Anaemia, Functional genomics

## Abstract

Hemoglobinopathies are the most common inherited anemias worldwide. While most carriers are asymptomatic or mildly affected, severe forms of α-thalassemia, including severe hemoglobin H (HbH) disease and surviving individuals with Hb Bart’s hydrops fetalis syndrome (BHFS), require lifelong transfusion support or stem cell transplantation. Reactivating the silenced embryonic α-like globin (ζ-globin) gene is a promising approach for treating these diseases. Here we identify the key *cis*-regulatory elements responsible for silencing the ζ-globin gene in definitive erythroid cells and subsequently develop gene-editing strategies that reactivate ζ-globin expression to therapeutically relevant levels. In a preclinical mouse model of BHFS, we show that this strategy rescues the otherwise prenatal lethal condition until late in development (E17.5). Using primary erythroid cells derived from patients with HbH disease and BHFS, we show that these approaches reactivate ζ-globin expression to levels exceeding those achieved in the mouse model, producing therapeutic levels of α-like globin.

## Abstract (web summary)

Liu et al. identify *cis*-regulatory elements that silence the embryonic ζ-globin gene in definitive erythroid cells. Gene-editing strategies reactivate ζ-globin expression to therapeutically relevant levels in mouse and human disease models.

## Main

Adult hemoglobin (HbA) is a tetramer composed of two α-globin and two β-globin chains (α_2_β_2_). Reduced or absent α-globin production causes α-thalassemia, one of the most common inherited disorders worldwide. Unaffected individuals have four α-globin genes (αα/αα), whereas α-thalassemia carriers have three (-α/αα) or two (--/αα or -α/-α) functional α-globin genes. In many areas of the world, the -α/ and --/ alleles are found at very high frequencies (up to ~70%) due to their selective advantage in the presence of malaria^1,2^. In these areas, compound heterozygotes (--/-α) have mild to moderate anemia, associated with excess nonfunctional β_4_ tetramers (hemoglobin H (HbH)), referred to as HbH disease. Approximately 10% of patients with HbH disease develop a transfusion-dependent phenotype^3^. Homozygotes who inherit no functional α-globin genes (--/--) have the most severe manifestation of α-thalassemia, called Hb Bart’s hydrops fetalis syndrome (BHFS). Individuals with BHFS produce nonfunctional tetramers of fetal β-like globin (γ_4_, Hb Bart’s), leading to profound anemia and usually intrauterine death during the second or third trimester of pregnancy. Despite this, over the past 40 years, due to improvements in intrauterine interventions and perinatal intensive care, a small number of neonates with BHFS who survived until late pregnancy have been salvaged and subsequently maintained into adulthood, albeit with lifelong transfusion dependence^4^. More recently, infants diagnosed early in pregnancy have survived because of intrauterine blood transfusions^5^. Currently, the only long-term curative treatment for patients with BHFS and transfusion-dependent HbH disease is hematopoietic stem cell transplantation. However, this approach is limited to a minority of patients with suitable human leukocyte antigen-matched donors, and, even when available, risks of graft-versus-host disease and transplant-related mortality remain^6^.

The human α-globin locus on chromosome 16 is one of the most thoroughly characterized mammalian loci. It comprises an embryonic α-like globin gene (ζ-globin gene, *HBZ*) and two fetal/adult α-globin genes (*HBA2* and *HBA1*), regulated by a cluster of enhancers (termed R1, R2, R3 and R4). These elements occur in the order of 5′-R1-R2-R3-R4-ζ-α2-α1-3′. The majority of patients with HbH disease and BHFS inherit the most common deletional allele, referred to as the Southeast Asian deletion (--^SEA^), in which a ~19.3 kb deletion removes both α-globin genes while leaving the enhancers and ζ-globin gene intact (5′-R1-R2-R3-R4-ζ-3′). It has previously been shown that ζ-globin contributes to hemoglobin tetramers termed Hb Portland 1 (ζ_2_γ_2_) and Hb Portland 2 (ζ_2_β_2_) that can functionally substitute for fetal (HbF, α_2_γ_2_) and adult (HbA, α_2_β_2_) forms of hemoglobin^7^. However, ζ-globin is normally completely silenced by 6–7 weeks of human gestation as the first (primitive) wave of erythropoiesis is replaced by the second and third (definitive) waves of hematopoiesis. Throughout fetal and adult life, the ζ-globin gene remains silenced. Rare spontaneous survivors of BHFS are thought to persistently express low levels of ζ-globin, suggesting that substantial reactivation of the ζ-globin gene would ameliorate the severity of this disease^4,8^. Therefore, more fully reactivating ζ-globin expression in definitive erythroid cells could offer a potential cure for transfusion-dependent α-thalassemia, in a situation analogous to individuals with increased HbF having reduced symptoms of β-haemoglobinopathies^9^. However, at present, the *cis*-regulatory elements and *trans*-acting factors responsible for transcriptional silencing of the ζ-globin gene are poorly understood.

Here using a genetically modified mouse model, we have investigated the DNA sequences flanking the ζ-globin gene and found that the *cis*-regulatory elements and associated chromatin environment required to silence ζ-globin expression are confined to a 4.4-kb region encompassing the ζ-globin gene. In previous studies, we and others have identified *trans*-acting pathways regulating ζ-globin expression^10–13^. In this study, by saturation mutagenesis of the ζ-globin gene promoter, we have identified discrete *cis*-regulatory elements that recruit repressive *trans*-acting factors in definitive erythroblasts. We found that mutating individual elements has a moderate effect on ζ-globin reactivation; however, a combined disruption reactivates ζ-globin expression to levels that would therapeutically benefit patients with severe forms of α-thalassemia. In addition to rescuing the severe phenotypes of α-thalassemia, this study reinforces the general principle that deleterious mutations in genes expressed in adults may be rescued by reactivating developmentally silenced functional paralogs.

## Results

### Developmental silencing of the ζ-globin gene is encoded within a 4.4-kb region that contains the gene

**Fig. 1:**
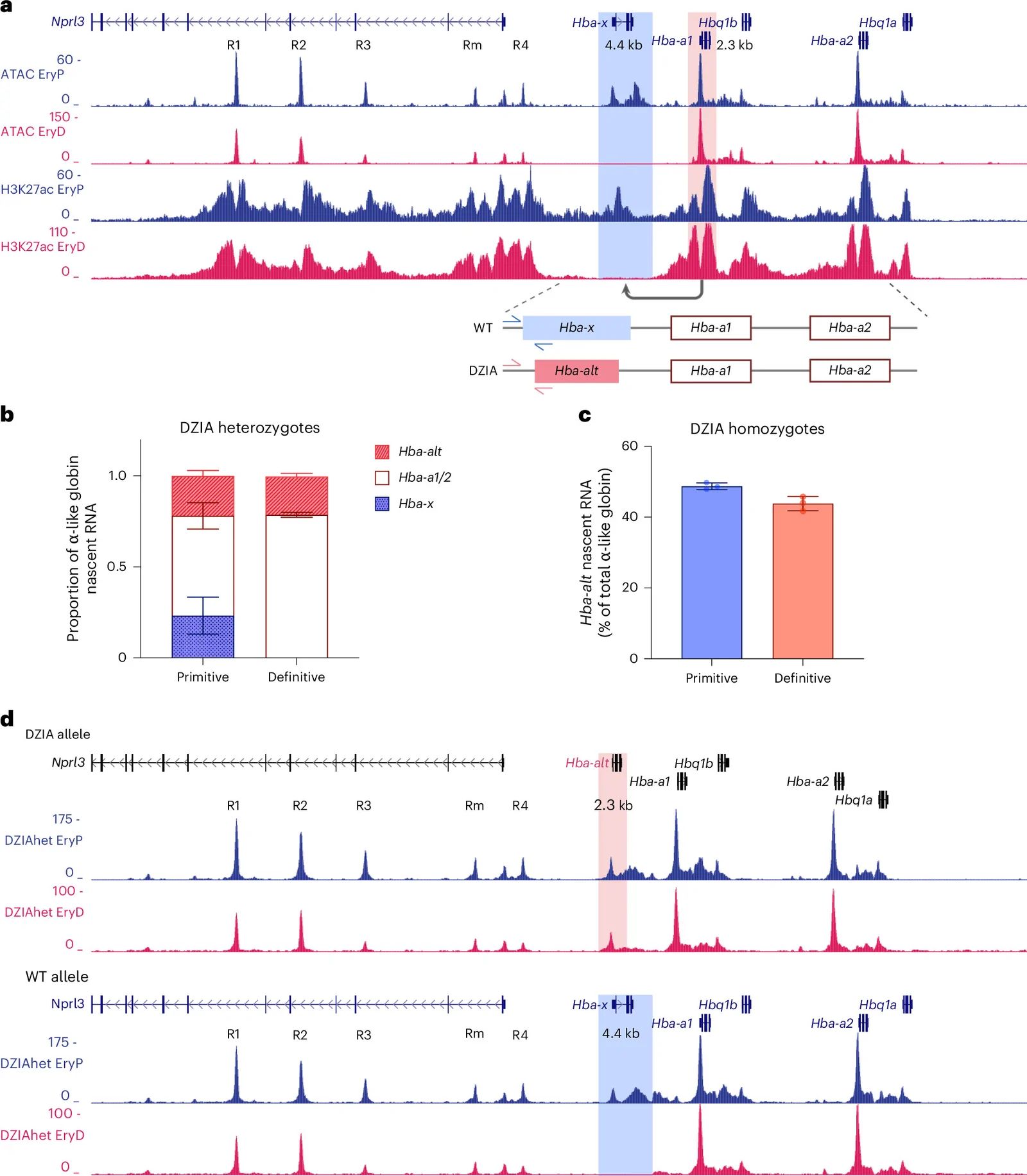
Chromatin environment and α-like globin expression in the DZIA mouse model. **a**, ATAC–seq and H3K27ac ChIP–seq in primitive (EryP, E10.5 blood) and definitive (EryD, E12.5 fetal liver) erythroblasts across the mouse α-globin locus (mm10, chr11: 32,234,000–32,314,000). The α-globin enhancers (R1–R4, Rm) are indicated. The blue highlight indicates the 4.4-kb region replaced by the modified α-globin (*Hba-alt*) gene in the DZIA model, and the gray arrow from *Hba-a1* to *Hba-x* indicates the replacement. The diagram below shows the resulting DZIA allele, and the harpoon arrows indicate the genotyping primer pairs used to distinguish the DZIA and WT alleles. **b**,**c**, Nascent RNA quantification of α-like globin genes in primitive (E10.5 blood) and definitive (E12.5 fetal liver) erythroid cells isolated from heterozygous (**b**) and homozygous (**c**) DZIA mouse embryos (*n* = 3 biological replicates). Data are presented as mean ± s.d. **d**, ATAC–seq in primitive (EryP, E10.5 blood) and definitive (EryD, E12.5 fetal liver) erythroblasts from heterozygous DZIA mouse embryos across the modified (DZIA, top) and WT (bottom) α-globin locus (mm10, chr11: 32,234,000–32,314,000). Source data

The ζ-globin gene is expressed in mouse embryonic erythroid cells during primitive erythropoiesis, driven by the α-globin enhancers (termed R1, R2, R3, Rm and R4)^10,14^. However, despite its proximity to the enhancers, which remain fully active, the ζ-globin gene is silenced in fetal and adult definitive erythroid cells in both humans and mice^15,16^. We have previously shown that, in mouse definitive erythroblasts, the ζ-globin gene lies within the α-globin subtopologically associating domain (~65 kb) but does not contact the α-globin enhancers^10,17^. Consistent with this suppression, the ζ-globin gene lies within a discrete subdomain (~10 kb) of inaccessible, hypoacetylated chromatin in definitive erythroblasts as determined by assay for transposase-accessible chromatin with high-throughput sequencing (ATAC–seq) and H3K27ac ChIP–seq (Fig. 1a). Previous studies suggest that the ζ-globin gene is repressed by sequences within its promoter, gene body and flanking regions^18,19^. However, the precise sequences responsible for ζ-globin regulation have not been identified.

**Extended Data Fig. 1:**
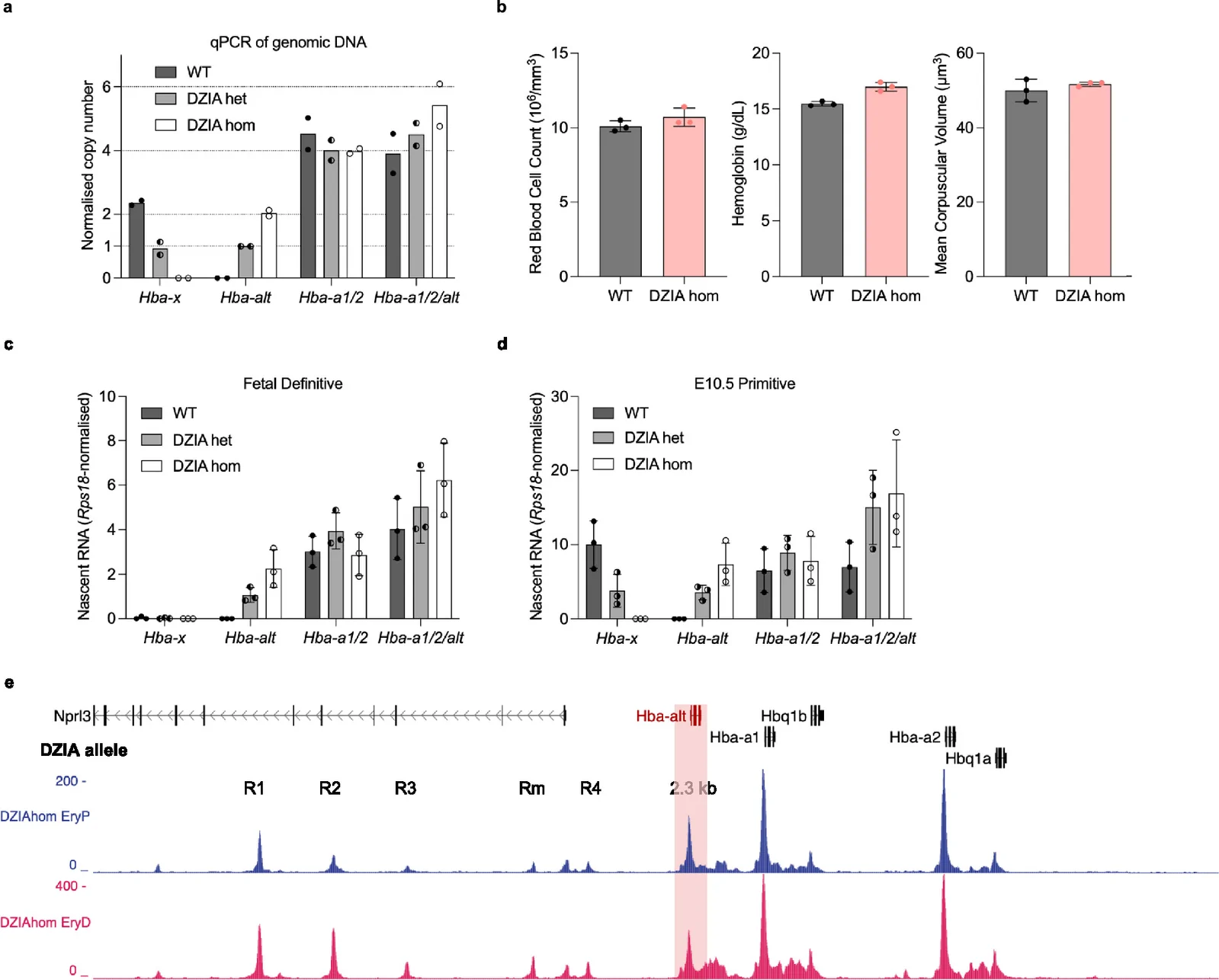
Characterization of the DZIA mouse model. **a**, Validation of qPCR primers for nascent RNA quantification using genomic DNA from WT and DZIA E12.5 fetal liver cells (*n* = 2 biological replicates). Data are presented as mean values. **b**, Hematological parameters of homozygous DZIA mice compared to WT mice (*n* = 3 male mice aged 19–21 weeks). Data are presented as mean ± s.d. **c**,**d**, Nascent RNA expression of α-like globin genes in fetal definitive (**c**) and E10.5 primitive (**d**) erythroid cells from DZIA mouse embryos (*n* = 3 biological replicates). Data are presented as mean ± s.d. **e**, ATAC–seq in primitive (EryP) and definitive (EryD) erythroblasts from homozygous DZIA mouse embryos across the modified (DZIA) α-globin locus (mm 10, chr11: 32,234,000–32,314,000). Pink highlight indicates the inserted *Hba-alt* gene at the position of the *Hba-x* gene.

To localize the *cis*-regulatory elements responsible for ζ-globin silencing, we examined a 4.4-kb segment containing the entire ζ-globin (*Hba-x*) gene in mice. This region is ATAC accessible in primitive erythroblasts and becomes inaccessible in definitive erythroblasts (Fig. 1a). We generated a mouse model in which this 4.4-kb region is replaced with a 2.3-kb fragment containing a modified α-globin (termed *Hba-alt*) gene that encodes 20 naturally occurring silent single-nucleotide polymorphisms, enabling to distinguish *Hba-alt* transcripts from the endogenous *Hba-a1*/*2* transcripts (Extended Data Fig. 1a). The inserted 2.3-kb segment corresponds to the region of open chromatin associated with the endogenous α-globin gene (Fig. 1a). This engineered mouse line was named deleted zeta inserted alpha (DZIA). Mice heterozygous and homozygous for this engineered allele are fully viable with normal hematological parameters (Extended Data Fig. 1b).

Of interest, we found that, in contrast to the normally silenced *Hba-x* gene, the substituted *Hba-alt* gene, now in the same relative position as the embryonic gene, is fully transcribed (Fig. 1b and Extended Data Fig. 1c) and ATAC accessible (Fig. 1d and Extended Data Fig. 1e) in definitive erythroblasts. In primitive erythroblasts, the *Hba-alt* gene is expressed at a level approximately equivalent to the endogenous *Hba-x* gene (Fig. 1b and Extended Data Fig. 1d). The levels of *Hba-alt* gene expression in primitive and definitive erythroblasts are comparable, accounting for 40–50% of total α-like globin nascent transcripts (Fig. 1c). Of note, expression of a single *Hba-alt* gene is roughly equivalent to the combined output of two endogenous α-globin genes. This increased level of expression may be due to its relative proximity to the α-globin enhancers. It is possible that the *Hba-alt* gene competes with endogenous α-globin genes for the enhancer activity, and its relative proximity to the enhancers places it in a more favorable position for frequent enhancer interactions. Together, these results indicate that the *cis-*regulatory elements and the associated chromatin environment responsible for ζ-globin silencing are encoded within the 4.4-kb region removed in the DZIA model, and neither the extended flanking sequences nor the gene’s position is involved in ζ-globin repression.

### Identification of repressive *cis*-regulatory elements within the mouse ζ-globin gene promoter

**Fig. 2:**
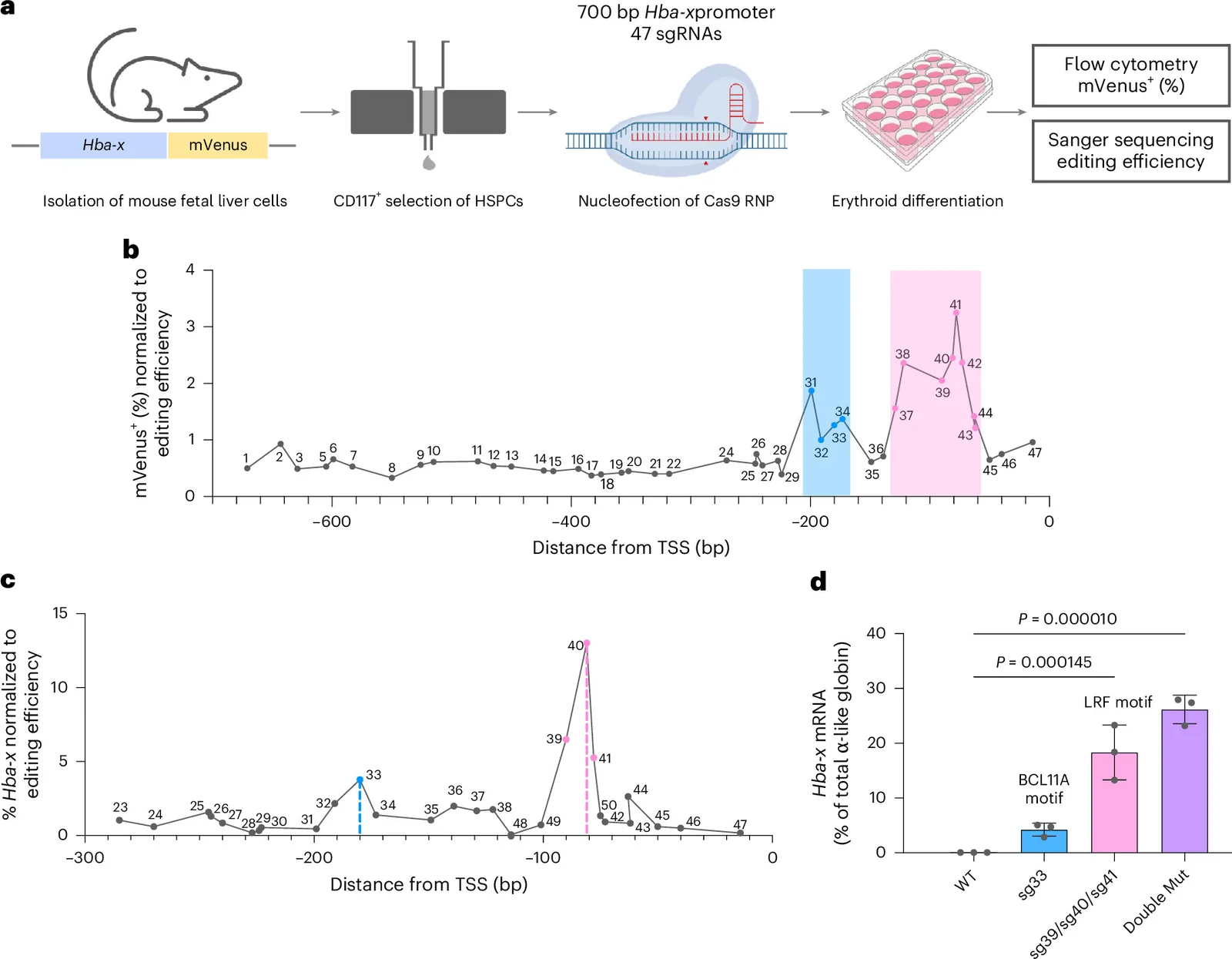
Identification of repressive *cis*-regulatory elements within the mouse ζ-globin gene promoter. **a**, Experimental design of the CRISPR–Cas9 screen across the mouse ζ-globin (*Hba-x*) gene promoter. **b**, Normalization of mVenus^+^ (%) to editing efficiency reveals two potential repressive regions, highlighted in blue and pink. **c**, Normalization of *Hba-x* mRNA (%) to editing efficiency in G1E-ER4 cells identifies two potential repressive *cis*-regulatory elements, targeted by sg33 and sg39/sg40/sg41. Data are presented as mean values (*n* = 2 biological replicates). **d**, Percentage of *Hba-x* mRNA to total α-like globin in clonal G1E-ER4 cell lines (3 independent single mutant clones, 3 independent replicates of a double-mutant clone). Data are presented as mean ± s.d. Exact *P* values are shown; statistical significance was assessed using one-way ANOVA with Dunnett correction for multiple comparisons. TSS, transcription start site; ANOVA, analysis of variance. Source data

**Extended Data Fig. 2:**
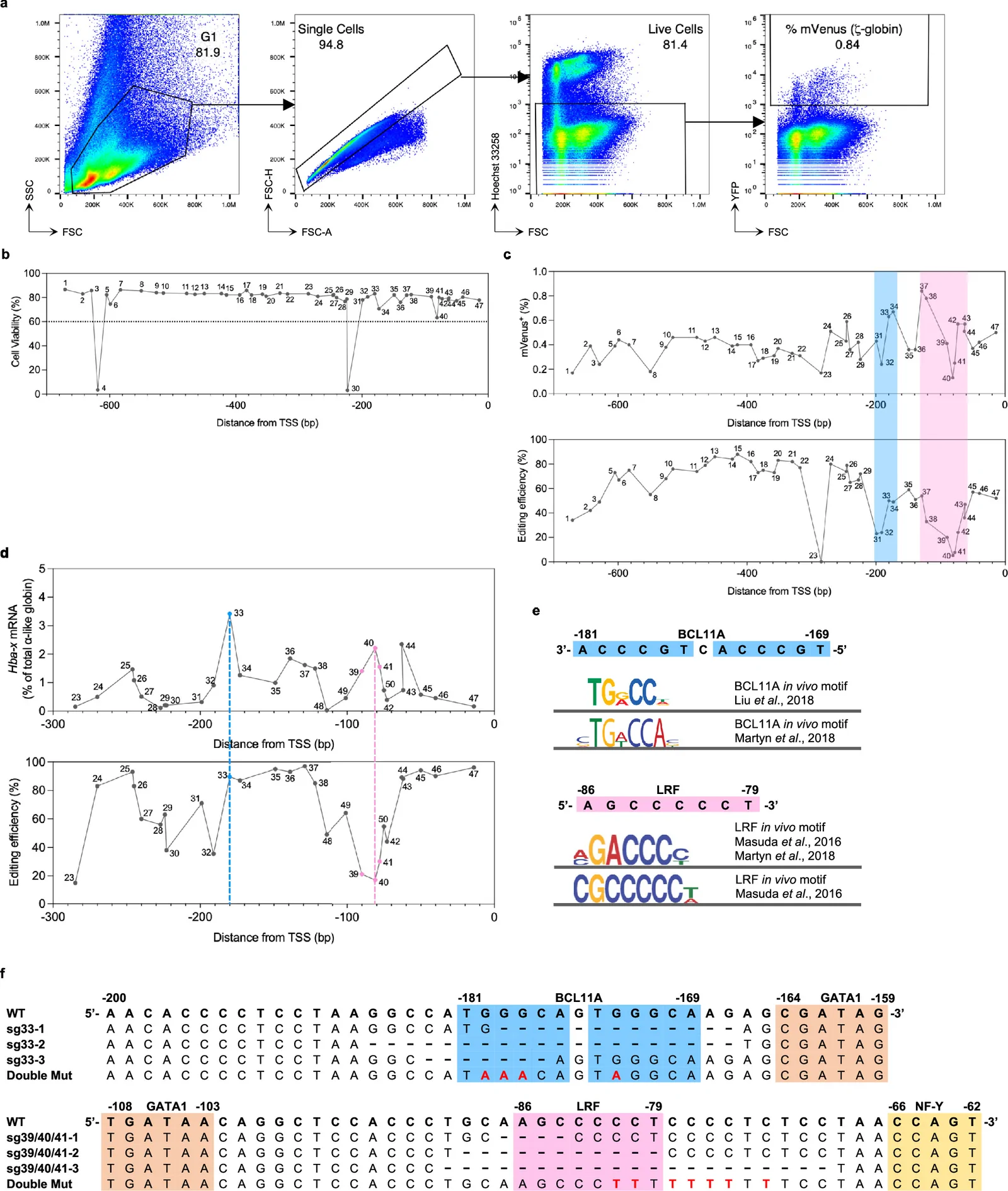
CRISPR screens identify repressive elements in the mouse ζ-globin gene promoter. **a**–**c**, Gating strategy (**a**) for experiment presented in Fig. 2a. Cell viability (**b**) mVenus^+^ (%) and editing efficiency (**c**) in fetal definitive erythroid cells from the CRISPR screen. **d**, Percentage of *Hba-x* mRNA to total α-like globin (upper) and sgRNA editing efficiency (lower) in G1E-ER4 cells from the CRISPR screen (*n* = 2 biological replicates). Data are presented as mean values. **e**, Identified repressive elements in the mouse *Hba-x* gene promoter align to BCL11A and LRF binding motifs from the listed references. **f**, DNA sequences of the *Hba-x* gene promoter of G1E-ER4 clonal cell lines. Dashes indicate deletions, and converted bases are shown in red.

Previous studies have suggested that the regulation of embryonic-specific genes is promoter centric^20^ and the ζ-globin promoter contains repressive *cis*-regulatory elements^18,21^. To identify these elements, we conducted a CRISPR/Cas9 screen of the ζ-globin gene promoter (Fig. 2a) using a mouse model in which exon three of the ζ-globin (*Hba-x*) gene has been replaced with a sequence encoding the fluorescence protein mVenus^10^. Fetal liver cells were obtained from heterozygous embryos at embryonic day 12.5 (E12.5) and cultured for up to 7 days. Hematopoietic stem and progenitor cells (HSPCs) were obtained by positive selection for CD117. Using SpCas9, we designed 47 sgRNAs (Methods) tiled along 700 bp 5′ of the *Hba-x* gene transcription start site, averaging one sgRNA for every 15 bp. CD117^+^ HSPCs were transfected with each Cas9/sgRNA RNP individually and differentiated into erythroid cells. Cell viability and mVenus (ζ-globin) expression were analyzed by flow cytometry, and chromatogram analysis using Inference of CRISPR Edits (Methods) was used to quantify the editing efficiency of each sgRNA (Extended Data Fig. 2a–c). sgRNAs that resulted in reduced cell viability (sg4 and sg30) and no detectable editing (sg23) were excluded from further analysis. Normalizing mVenus^+^ (%) to editing efficiency revealed two regions in the proximal 200 bp of the *Hba-x* gene promoter, which, when perturbed, upregulate ζ-globin expression (Fig. 2b).

To identify the precise sequences involved, we further interrogated the proximal 300 bp of the *Hba-x* gene promoter with sg23–sg47 and three additional sgRNAs (sg48, sg49, sg50; Methods) in an immortalized mouse proerythroblast cell line termed G1E-ER4 (ref. ^22^), in which higher editing efficiencies were achieved than in primary cells (Extended Data Fig. 2d). After erythroid induction, the percentage of *Hba-x* mRNA of total α-like globin (Extended Data Fig. 2d) was assessed by TaqMan Gene Expression assay, which is more sensitive than detecting protein level. With these improvements, we identified four sgRNAs that increase ζ-globin mRNA (Fig. 2c) and found that they target the predicted binding motifs of BCL11A (sg33) and ZBTB7A (also known as LRF; sg39, sg40, sg41), respectively (Extended Data Fig. 2e). Of interest, we and others have previously shown that reducing the levels of *trans*-acting factors BCL11A and LRF alone and in combination increases ζ-globin expression in both human and mouse definitive erythroid cells^10,23^.

After the CRISPR screen, clonal G1E-ER4 cell lines harboring small (4–21 bp) biallelic deletions (Extended Data Fig. 2f) at the two identified motifs were generated to determine the exact level of ζ-globin derepression. Disruptions of the predicted BCL11A and LRF binding motifs derepress ζ-globin transcription accounting for ~4% and ~18% of total α-like globin, respectively (Fig. 2d). To determine the combined effect of these two elements, a cytosine base editor (CBE) was used to introduce mutations at both sites simultaneously (Methods), and a clonal cell line with double mutations was generated (Extended Data Fig. 2f). The double-mutant cells show a higher (~26%) ζ-globin upregulation (Fig. 2d), suggesting a combined effect that is marginally greater than the sum of its parts. It is worth noting that the single-mutant and double-mutant cell lines were generated using different methods and therefore may not be strictly comparable.

### ζ-globin reactivation in a preclinical model in vivo

**Fig. 3:**
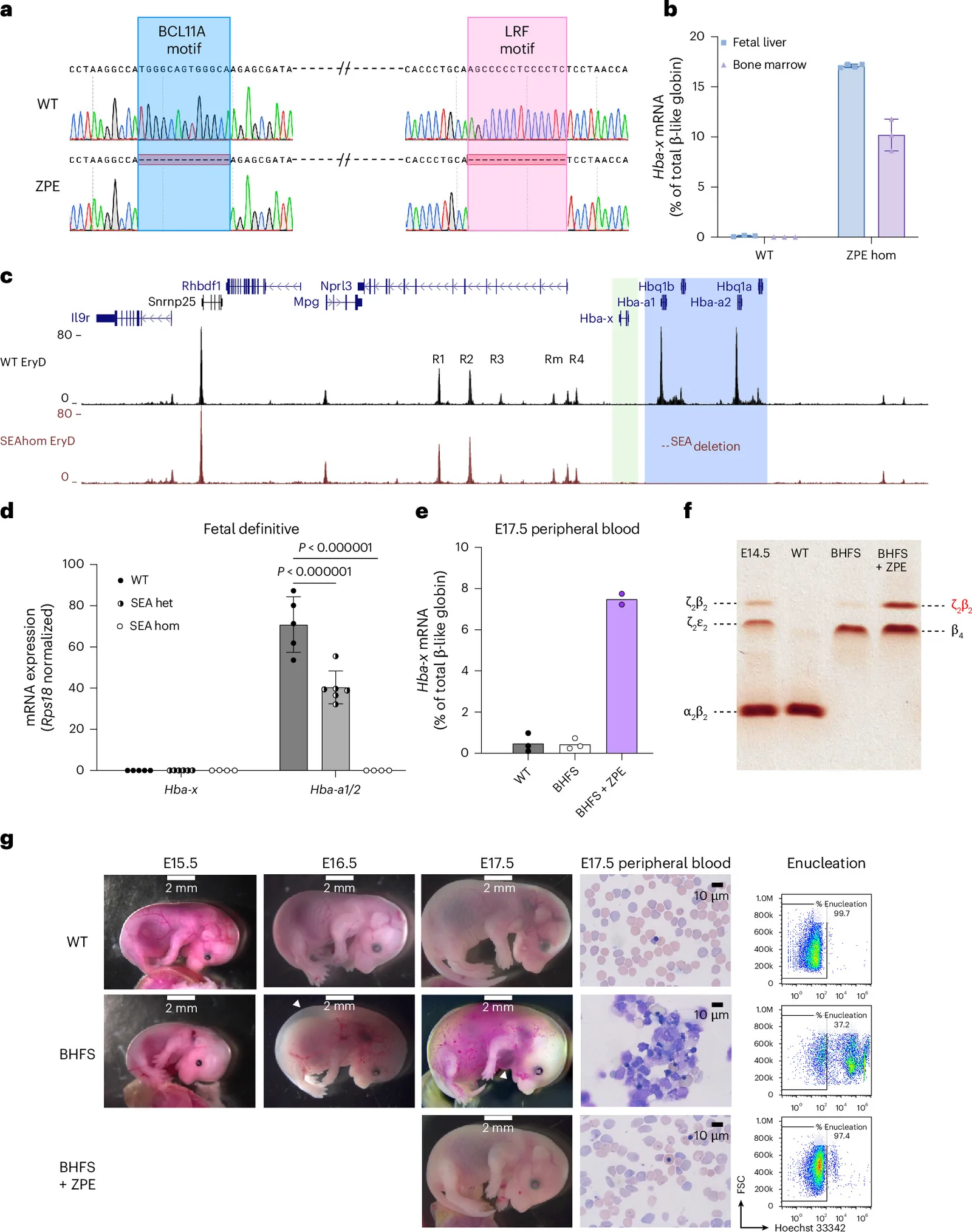
ζ-globin promoter edits ameliorate the lethal α-thalassemia phenotype in vivo. **a**, Sanger sequencing results showing the *Hba-x* gene promoter sequence in the ZPE mouse model. Deletions of BCL11A and LRF motifs are indicated. **b**, Percentage of *Hba-x* mRNA to total β-like globin in E14.5 fetal liver cells and Ter119^+^ bone marrow cells from WT and ZPE homozygotes (*n* = 4 ZPE homozygous E14.5 embryos, *n* = 3 biological replicates for other groups). Data are presented as mean ± s.d. Bone marrow cells were isolated from male mice aged 13–15 weeks. **c**, ATAC–seq across the α-globin locus (mm10, chr11: 32,185,000–32,329,000) in fetal definitive erythroblasts from E12.5 WT and BHFS mouse embryos. The *Hba-x* gene is highlighted in green, the --^SEA^ deletion is highlighted in blue. The α-globin enhancers (R1–R4, Rm) are indicated. **d**, α-like globin mRNA expression in the WT, heterozygous and homozygous --^SEA^ deletion mouse models (*n* = 5, 6 and 4 biological replicates, respectively). Data are presented as mean ± s.d. *P* values are shown; statistical significance was assessed using two-way ANOVA with Dunnett correction for multiple comparisons. **e**, Percentage of *Hba-x* mRNA to total β-like globin in peripheral blood from WT, BHFS and BHFS + ZPE E17.5 embryos (*n* = 3, 3 and 2 biological replicates, respectively). Data are presented as mean values. **f**, Isoelectric focusing of extracts from E17.5 peripheral blood of WT, BHFS and BHFS + ZPE embryos. Extracts from E14.5 peripheral blood (lane 1) are used as mouse hemoglobin markers and positive controls for Hb Portland 2 (ζ_2_β_2_). Experiments were independently repeated thrice with similar results. **g**, Representative microscopy of WT and BHFS mouse embryos at E15.5–E17.5, white triangles indicate signs of edema. Scale bar = 2 mm. Next to the embryos, representative cytospins of peripheral blood from E17.5 embryos stained using a modified Wright’s procedure. Scale bar = 10 μm. Right, flow cytometry analysis of erythroid enucleation of peripheral blood from E17.5 embryos. Enucleated cells are identified as Hoechst 33342-negative cells. FSC, forward scatter. M, million. Similar results were obtained from at least two independent embryos. Source data

**Extended Data Fig. 3:**
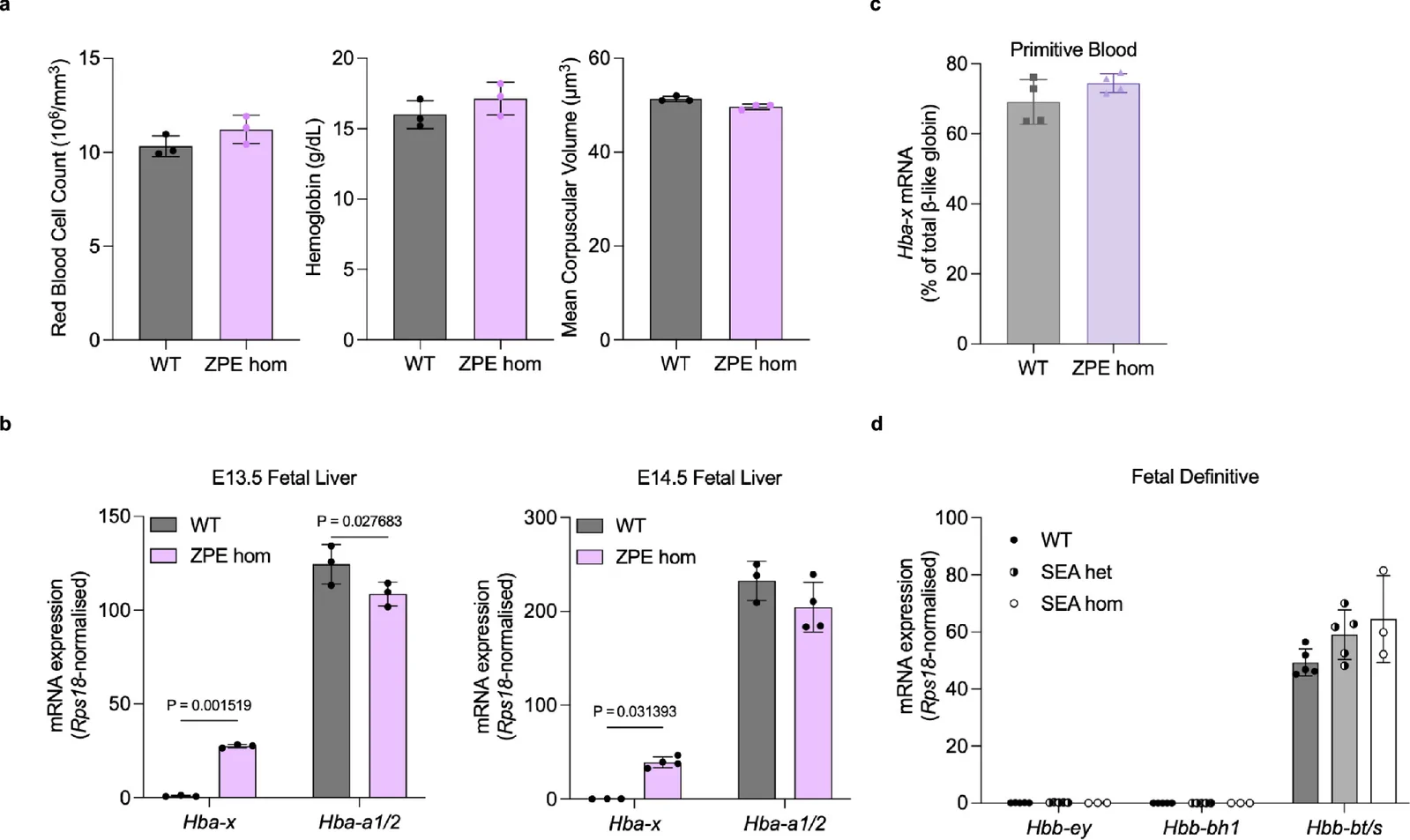
Charaterization of the ZPE and SEA deletion mouse models. **a**, Hematological parameters of homozygous ZPE mice compared to WT mice (*n* = 3 male mice aged 13–15 weeks). Data are presented as mean ± s.d. **b**, α-like globin mRNA expression in fetal liver cells from WT and ZPE homozygous embryos at E13.5 and E14.5 (n = 4 ZPE homozygous E14.5 embryos, *n* = 3 embryos for other groups). Data are presented as mean ± s.d. Exact *P* values are shown in the figure; statistical significance was assessed using two-way ANOVA with Bonferroni correction for multiple comparisons. **c**, Percentage of *Hba-x* mRNA to total β-like globin in primitive blood from WT and ZPE homozygous E11.5-E12.5 embryos (*n* = 4 biological replicates). **d**, β-like globin mRNA expression in the WT, heterozygous and homozygous --^SEA^ deletion mouse models (*n* = 5, 5 and 3 biological replicates, respectively). Data are presented as mean ± s.d.

After the identification and validation of two repressive *cis*-regulatory elements in the ζ-globin promoter, we further tested the effect of these edits in vivo. Combined with CRISPR/Cas9, a donor DNA harboring deletions of the two identified elements (27 bp deletion in total) was used to generate a mouse model with a modified ζ-globin gene promoter, termed ζ-globin promoter edited (ZPE; Fig. 3a). Mice heterozygous and homozygous for this modified allele are fully viable with normal hematological parameters (Extended Data Fig. 3a). In homozygous ZPE mice, ζ-globin expression is reactivated in both fetal and adult definitive erythroid cells, accounting for ~17% and ~10% of total β-like globin mRNA, respectively (Fig. 3b and Extended Data Fig. 3b). While in primitive erythroid cells, ζ-globin expression is unaffected (Extended Data Fig. 3c). These data suggest that the promoter edits effectively reactivate ζ-globin expression in definitive erythroid cells in vivo without impairing normal erythropoiesis or development.

To assess whether this level of ζ-globin expression is therapeutic in a preclinical model, we generated a mouse line harboring a deletion equivalent to the --^SEA^ deletion in humans (Fig. 3c). Unlike previous models of BHFS, this model precisely replicates the naturally occurring --^SEA^ deletion, including flanking sequences and DNA structure, which could affect ζ-globin expression. Heterozygous SEA mice are asymptomatic, mirroring the situation in human --^SEA^ carriers, expressing about half of the α-globin compared to wild type (WT; Fig. 3d). Heterozygous SEA mice were crossed to generate homozygous (--^SEA^/--^SEA^) embryos as a BHFS disease model, which has no α-globin (Fig. 3d) and normal β-like globin expression (Extended Data Fig. 3d). In definitive erythroblasts from BHFS mouse embryos, the ζ-globin gene remains silenced (Fig. 3d,e) and ATAC inaccessible (Fig. 3c).

Before E15.5, BHFS embryos are indistinguishable from their WT littermates by appearance, because a small proportion of primitive erythroid cells expressing ζ-globin are still in circulation. However, from E15.5 when primitive erythroid cells disappear, BHFS embryos become smaller and paler than their WT littermates, broken blood vessels are visible, and these phenotypes become more pronounced during the subsequent developmental days (Fig. 3g). By E16.5, signs of edema are present; by E17.5, the embryos suffer from severe hemolytic anemia and hydrops fetalis (Fig. 3g). Blood cytospins and flow cytometry analysis show a large proportion of nucleated immature erythroblasts (Fig. 3g) producing nonfunctional β_4_ (Fig. 3f) in E17.5 blood from BHFS embryos, suggesting impaired erythroid maturation caused by α-globin deficiency. These abnormalities faithfully recapitulate the conditions seen in human BHFS fetuses^9^.

By introducing the ζ-globin promoter edits to the --^SEA^ allele using the same strategy as generating the ZPE mouse model, we were able to obtain BHFS embryos with the ζ-globin promoter edits (BHFS + ZPE). These ‘ζ-globin only’ embryos show dramatic improvements compared to BHFS embryos at E17.5, with near-normal enucleated red blood cells (Fig. 3g) expressing ~7.5% ζ-globin mRNA (Fig. 3e) and producing Hb Portland 2 (ζ_2_β_2_; Fig. 3f). These data suggest that ζ-globin may functionally substitute for α-globin to ameliorate severe α-thalassemia in vivo. Together, these results show that editing the identified repressive *cis*-regulatory elements within the ζ-globin promoter has translational potential in patients with severe forms of α-thalassemia.

### Identifying and base editing the corresponding elements in the human ζ-globin gene promoter

**Fig. 4:**
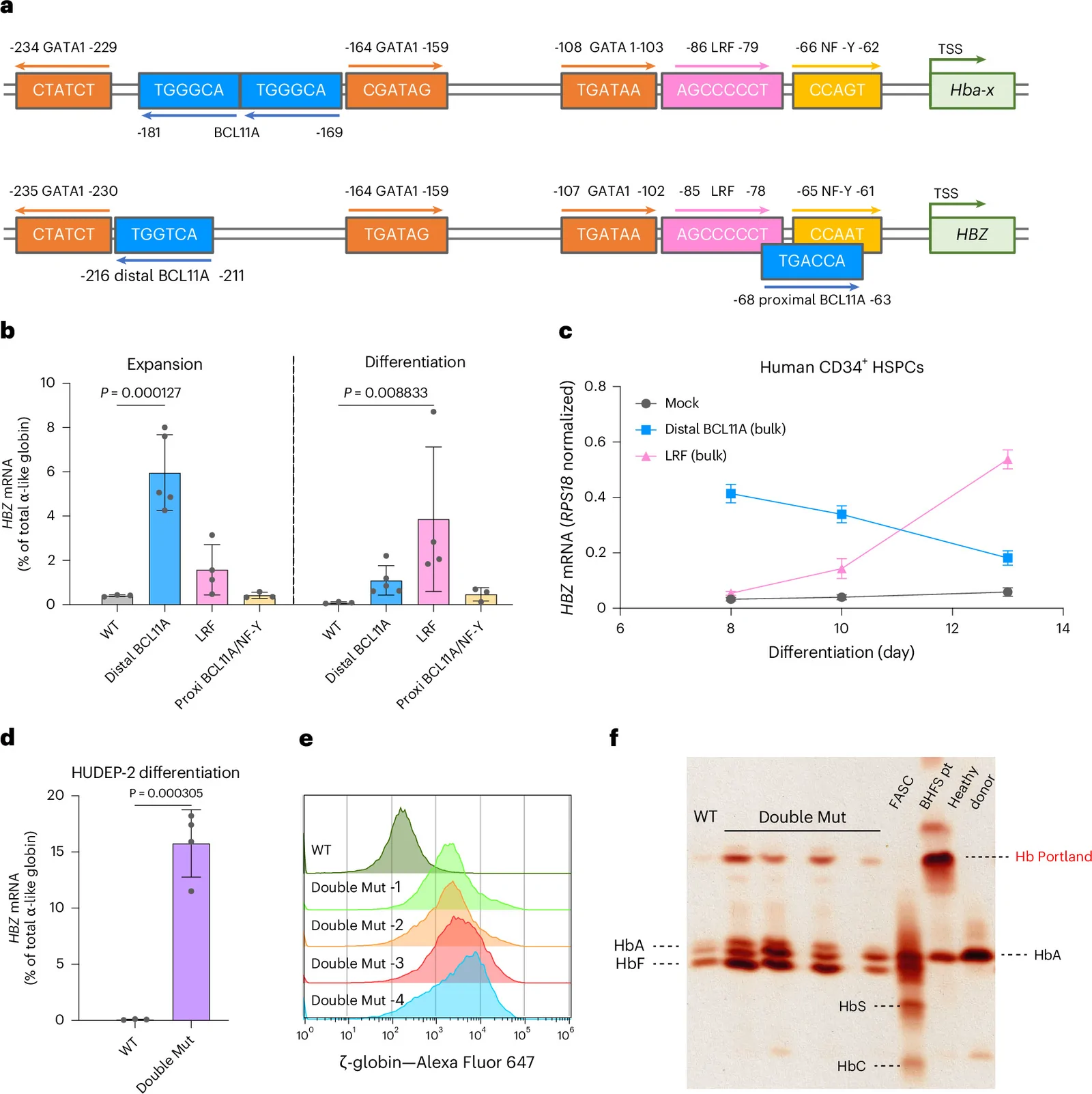
Verifying the corresponding repressive motifs in the human ζ-globin gene promoter. **a**, Schematic structures of the ζ-globin gene promoter of mouse (*Hba-x*, top) and human (*HBZ*, bottom). Binding motifs for BCL11A, LRF, GATA1 and NF-Y are indicated. **b**, Percentage of *HBZ* mRNA to total α-like globin in WT and mutant HUDEP-2 cell lines (*n* = 3 biological replicates for WT, *n* = 5, 4 and 3 independent clones for distal BCL11A motif mutant, LRF motif mutant and proximal BCL11A motif mutant, respectively) before and after differentiation (day 8). Data are presented as mean ± s.d. Exact *P* values are shown; statistical significance was assessed using two-way ANOVA with Dunnett correction for multiple comparisons. **c**, Quantification of *HBZ* mRNA during erythroid differentiation of CD34^+^ HSPCs, base-edited cells are in bulk populations (*n* = 3 biological replicates). Data are presented as mean ± s.d. **d**, Percentage of *HBZ* mRNA to total α-like globin in clonal HUDEP-2 cell lines with mutations at both the distal BCL11A and the LRF binding sites (double mutant, *n* = 4 independent clones). WT cells are presented as controls (WT, *n* = 3 biological replicates). Data are presented as mean ± s.d. Exact *P* value is shown; statistical significance was assessed using an unpaired two-tailed *t* test. **e**, Intracellular staining using Alexa Fluor 647-labeled ζ-globin antibody shows that double-mutant HUDEP-2 cell lines produce ζ-globin protein in a pan-cellular manner. **f**, Isoelectric focusing of extracts from WT (lane 1) and four independent double-mutant HUDEP-2 clones (lanes 2–5) on day 10 of differentiation, showing production of hemoglobins. An FASC control (lane 6), extracts of blood from a transfused BHFS patient (lane 7) and a healthy donor (lane 8) are used as markers. Hemoglobin molecules are indicated. Source data

**Extended Data Fig. 4:**
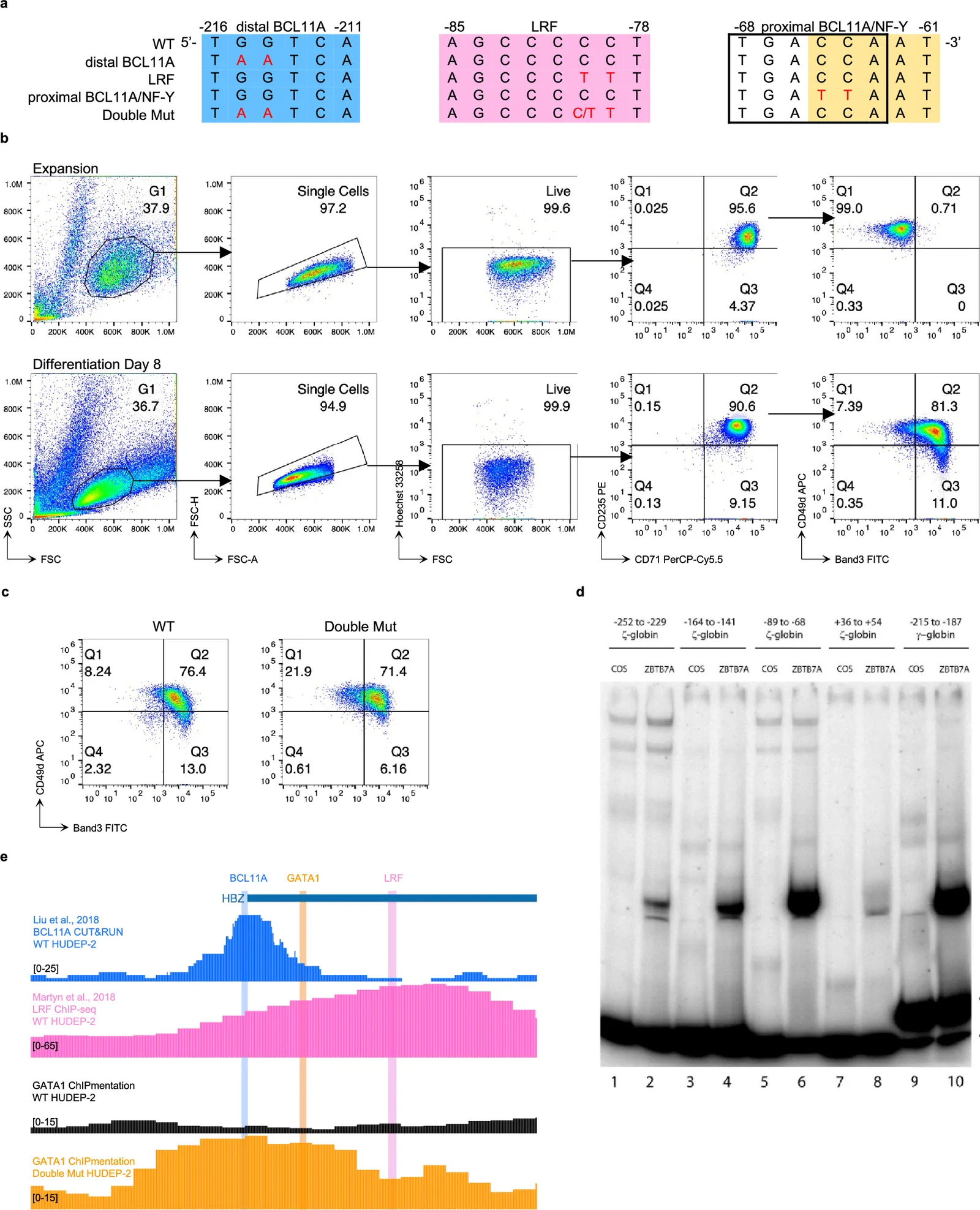
Identification of the repressive elements in the human ζ-globin gene promoter. **a**, DNA sequences of the *HBZ* gene promoter of HUDEP-2 clonal cell lines. Converted bases are shown in red. **b**, Gating strategy of flow cytometry analysis of HUDEP-2 cells, the upper panel shows undifferentiated cells in expansion medium, the lower panel shows cells on day 8 of erythroid differentiation. **c**, Representative flow cytometry analysis of wild-type (WT) and double mutant (Double Mut) HUDEP-2 cells on day 8 of differentiation. **d**, EMSA showing strong binding of LRF (ZBTB7A) to the oligonucleotides representing the human ζ-globin promoter, particularly to sites –89 to –68 (lane 6). Lane 10 is a positive control, showing LRF (ZBTB7A) binds to the γ-globin –200 site. **e**, Transcription factor binding at the human ζ-globin gene promoter (hg38, chr16: 152,450–152,900). The top two tracks are CUT&RUN and ChIP–seq data for BCL11A and LRF binding in WT HUDEP-2 cells from the listed publications. The bottom two tracks are GATA1 ChIPmentation data from WT and double mutant HUDEP-2 cells. The binding motifs are highlighted and indicated.

Direct binding of BCL11A and LRF to the human ζ-globin (*HBZ*) gene promoter has been shown^23–25^ in the HUDEP-2 cell line, a human definitive erythroid cell line^26^. In addition, motif analysis of the *HBZ* gene promoter reveals their likely binding motifs (Fig. 4a). The LRF motif is highly conserved in mice and humans and bound by LRF protein, as shown in the electrophoretic mobility shift assay (Extended Data Fig. 4d). By contrast, we noted divergence in the sequence and position of putative BCL11A motifs. In the human *HBZ* gene promoter, two consensus BCL11A motifs lie 148 bp apart, with the proximal site overlapping the CCAAT box (NF-Y binding motif; Fig. 4a). Previous footprint analysis of CUT&RUN data indicates that BCL11A binds to the distal site in human CD34^+^ HSPCs^25^.

To investigate the role of these elements within the human *HBZ* gene promoter, we examined the HUDEP-2 cell line, which has a very low background of ζ-globin expression in expansion culture and in erythroid differentiation (Fig. 4b). CBEs were used to introduce mutations (Methods) and avoid creating large deletions between the *HBZ* gene and the almost identical *HBZP1* pseudogene. Independent HUDEP-2 cell clones with mutations at BCL11A and LRF motifs were generated (Extended Data Fig. 4a) and differentiated into erythroid cells, monitored by immunophenotyping (Extended Data Fig. 4b).

Globin gene expression in these clones before and after erythroid differentiation shows that disrupting the proximal BCL11A binding motif does not reactivate ζ-globin expression (Fig. 4b), most probably because its role cannot be separated from the overlapping CCAAT box, which is essential for gene transcription^27,28^. Mutations at the distal BCL11A binding site derepress ζ-globin expression during the expansion phase; however, this effect fades away in differentiated erythroid cells (Fig. 4b). Conversely, editing the LRF motif shows little effect in the expansion phase, but increases ζ-globin expression during erythroid differentiation (Fig. 4b). These effects were recapitulated during erythroid differentiation of primary human CD34^+^ HSPCs (Fig. 4c). Disruption of both the distal BCL11A binding site and the LRF binding site in HUDEP-2 cells (double mutant; Extended Data Fig. 4a) increases ζ-globin expression such that it accounts for ~15% of total α-like globin (Fig. 4d); this is comparable to the levels seen in cells null for both BCL11A and LRF^10^, suggesting these two *trans*-acting factors repress ζ-globin gene largely by direct promoter binding. In double-mutant cells, flow cytometry analysis of ζ-globin protein shows pan-cellular expression (Fig. 4e), and isoelectric focusing shows production of Hb Portland (Fig. 4f). Notably, in contrast to the transcription factor knockout, editing the binding motifs at the ζ-globin promoter does not affect erythroid differentiation (Extended Data Fig. 4c). Inactivating the repressive motifs enables GATA1 to bind the ζ-globin promoter (Extended Data Fig. 4e).

### Editing the ζ-globin gene promoter in a HUDEP-2 cell model of BHFS (--^SEA^/--^SEA^)

**Fig. 5:**
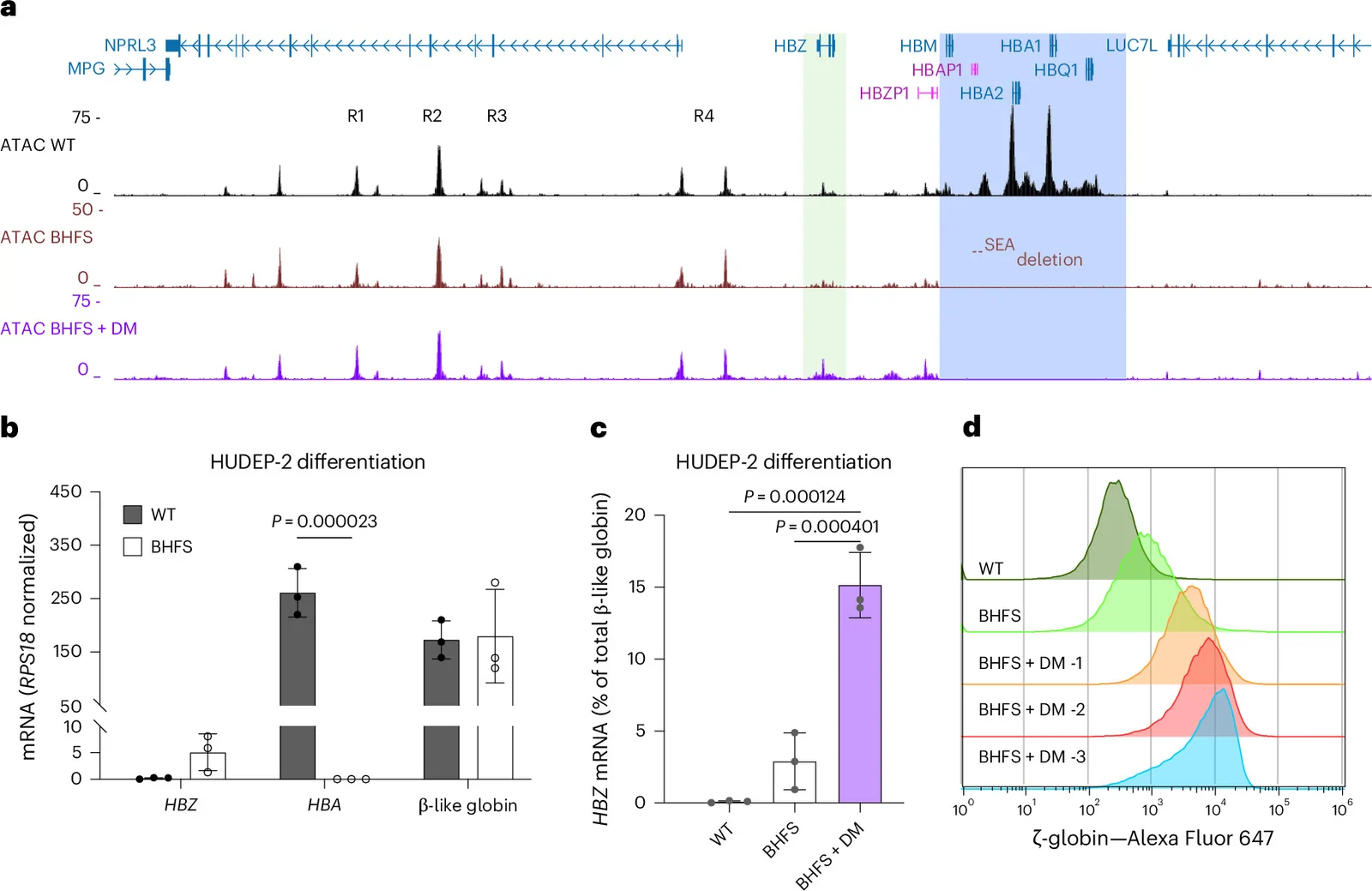
ζ-globin gene promoter editing in a BHFS HUDEP-2 cell model. **a**, ATAC–seq tracks across the human α-globin locus (hg38, chr16: 80,000–210,000) in HUDEP-2 cells on day 8 of differentiation. The ζ-globin (*HBZ*) gene is highlighted in green; the --^SEA^ deletion is highlighted in blue. **b**, mRNA quantification of globin genes in WT and BHFS HUDEP-2 cells on day 8 of differentiation (*n* = 3 biological replicates). Data are presented as mean ± s.d. Exact *P* value is shown; statistical significance was assessed using two-way ANOVA with Bonferroni correction for multiple comparisons. **c**, Percentage of *HBZ* mRNA to total β-like globin in WT, BHFS (*n* = 3 biological replicates) and clonal BHFS HUDEP-2 cell lines with mutations at both the distal BCL11A and the LRF binding sites (BHFS + DM, *n* = 3 independent clones). Data are presented as mean ± s.d. Exact *P* values are shown; statistical significance was assessed using one-way ANOVA with Bonferroni correction for multiple comparisons. **d**, Intracellular staining using Alexa Fluor 647-labeled ζ-globin antibody shows slightly increased ζ-globin protein expression in the BHFS HUDEP-2 cell model, and the promoter double mutation (BHFS + DM) further induces ζ-globin expression. Source data

Although in unaffected individuals (αα/αα) the ζ-globin gene is transcriptionally silenced at 6–7 weeks of gestation, in peripheral blood from carriers of the --^SEA^ deletion, there are persistent, albeit very low levels of ζ-globin expression^29–31^, and fetuses with BHFS (--^SEA^/--^SEA^) produce 10–20% Hb Portland 1 (refs. ^32–34^); the mechanism of the incomplete ζ-globin repression in this context is unclear. To better understand ζ-globin gene regulation in the --^SEA^ haplotype, and to test the ζ-globin promoter edits in a disease context, we generated a HUDEP-2 cell line homozygous for the most prevalent α^0^-thalassemia deletion (--^SEA^/--^SEA^), confirmed by gap-PCR and ATAC–seq (Fig. 5a).

**Extended Data Fig. 5:**
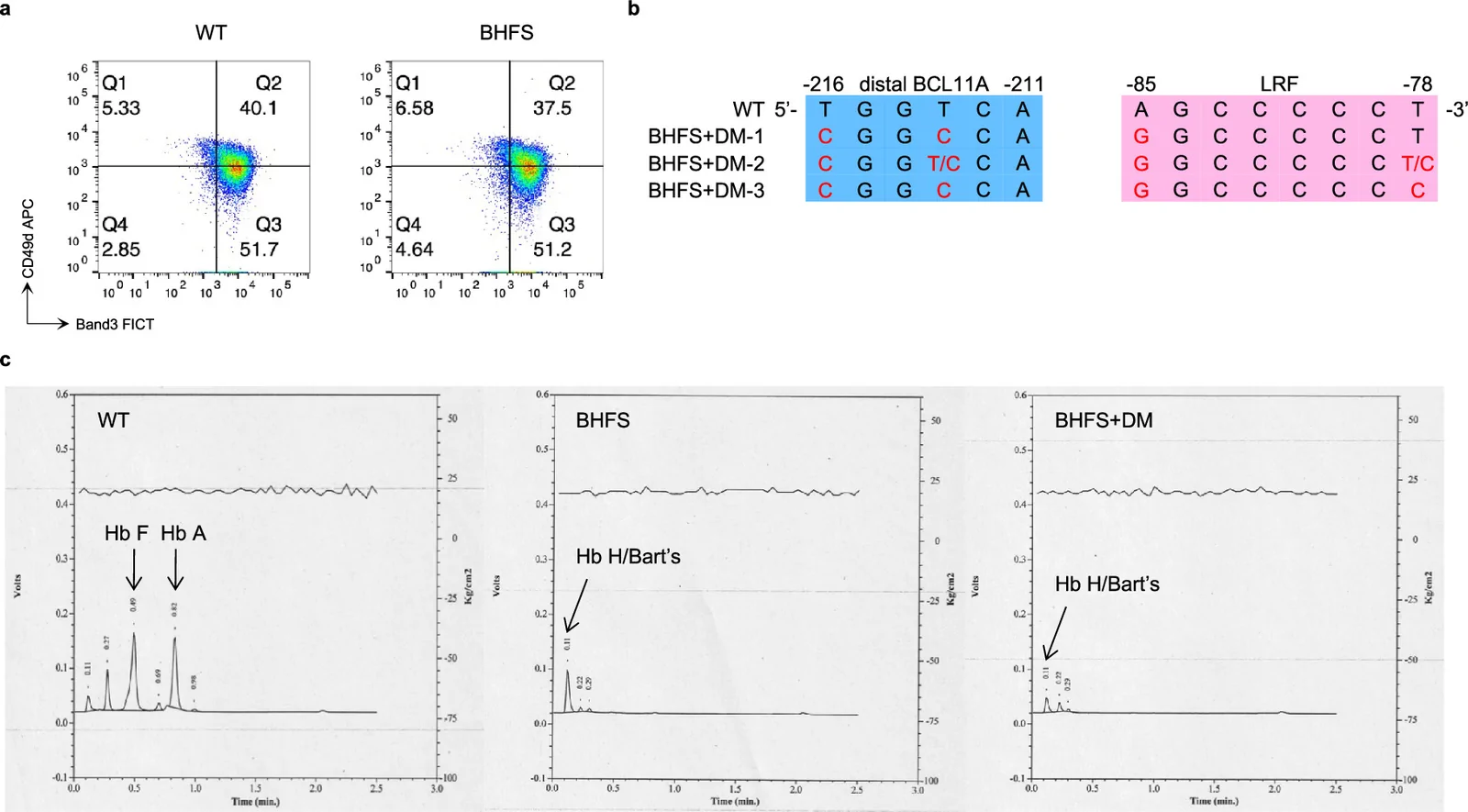
ζ-globin gene promoter editing in the BHFS HUDEP-2 cell model. **a**, Representative flow cytometry analysis of WT and BHFS HUDEP-2 cells on day 8 of differentiation. **b**, DNA sequences of the *HBZ* gene promoter of double mutant BHFS HUDEP-2 clones. Converted bases are indicated in red. **c**, HPLC analysis of WT, BHFS, and double mutant BHFS (BHFS + DM) HUDEP-2 cells on day 10 of differentiation. Peaks corresponding to hemoglobin tetramers are annotated.

As HUDEP-2 cells show limited enucleation efficiency in culture, and only enucleated erythroid cells show the hemolytic phenotype, this BHFS HUDEP-2 cell line can expand and differentiate normally with no α-globin but normal β-like globin expression (Fig. 5b and Extended Data Fig. 5a). Mirroring the situation seen in BHFS patients and the --^SEA^ deletion carriers, ζ-globin expression is slightly increased (Fig. 5b–d), but there is no obvious change in chromatin accessibility over the *HBZ* gene (Fig. 5a).

To test the effect of mutating both BCL11A and LRF binding sites within the ζ-globin promoter in the --^SEA^/--^SEA^ context, we further generated independent clonal BHFS cell lines with the desired mutations (BHFS + DM; Extended Data Fig. 5b) using adenine base editors (ABEs). Similarly, these mutations increase ζ-globin expression at both mRNA and protein levels (Fig. 5c,d). In this case, ATAC–seq data show increased chromatin accessibility over the *HBZ* gene (Fig. 5a). ζ-globin mRNA accounts for ~15% of total β-like globin in double-mutant BHFS erythroid cells (Fig. 5c), which is comparable to that seen with the ζ-globin promoter edits in the αα/αα context, suggesting the effect size of promoter edits is not noticeably affected by the --^SEA^ deletion in the HUDEP-2 cell model. Notably, HPLC analysis demonstrated that the double mutation was associated with reduced β-like globin tetramers (HbH/Bart’s) in BHFS HUDEP-2 cells (Extended Data Fig. 5c), providing functional evidence that ζ-globin can compensate for α-globin deficiency and ameliorate globin chain imbalance.

### ζ-globin reactivation in primary CD34^+^ HSPC-derived erythroid cells from patients with HbH disease and BHFS

**Fig. 6:**
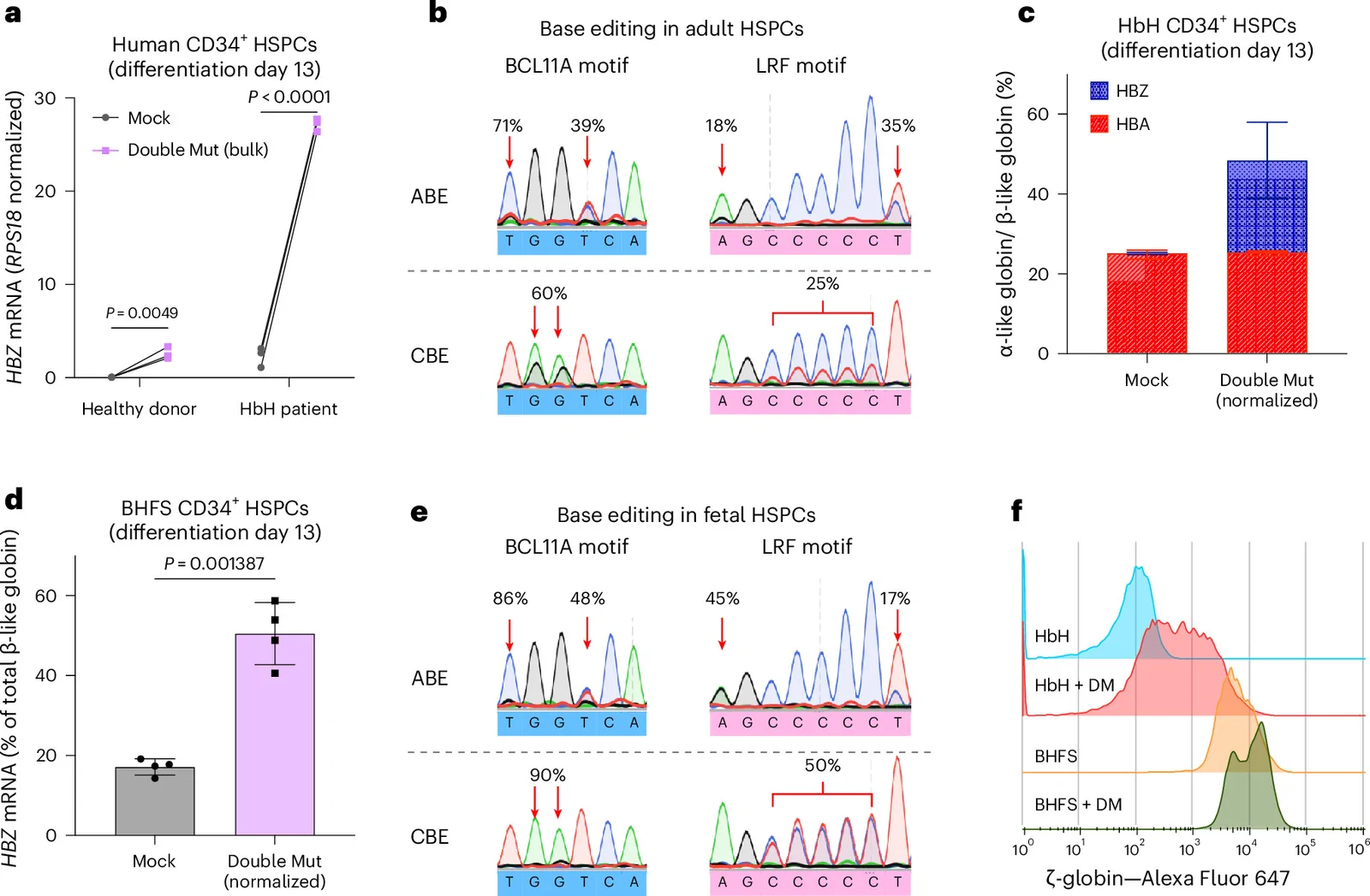
ζ-globin gene promoter editing in primary CD34^+^ HSPC-derived erythroid cells. **a**, Mutation of both repressive motifs (Double Mut) increases *HBZ* mRNA expression in CD34^+^ HSPC-derived erythroid cells from healthy donors and HbH patients (*n* = 3 biological replicates). *P* values are shown; statistical significance was assessed using two-way ANOVA with Bonferroni correction for multiple comparisons. **b**, Base-editing quantification using EditR shows representative editing efficiencies at the BCL11A and LRF motifs in adult HSPCs using ABE or CBE. **c**, Percentage of α-like globin to β-like globin in erythroid cells derived from HbH patients (*n* = 3 biological replicates), *HBZ* mRNA expression is normalized to editing efficiencies. Data are presented as mean ± s.d. **d**, Mutation of both repressive motifs increases *HBZ* mRNA in HSPC-derived erythroid cells from a 21-week BHFS fetus (4 independent experiments). Expression of BCL11A isoforms in these cells is unknown. Data are presented as mean ± s.d. Exact *P* value is shown; statistical significance was assessed using a paired two-tailed *t* test. **e**, Base-editing quantification using EditR shows representative editing efficiencies at the BCL11A and LRF motifs in fetal HSPCs. **f**, Intracellular staining using Alexa Fluor 647-labeled ζ-globin antibody shows increased ζ-globin protein expression in double-mutant erythroid cells (DM, in bulk) derived from HbH and BHFS patients. Source data

**Extended Data Fig. 6:**
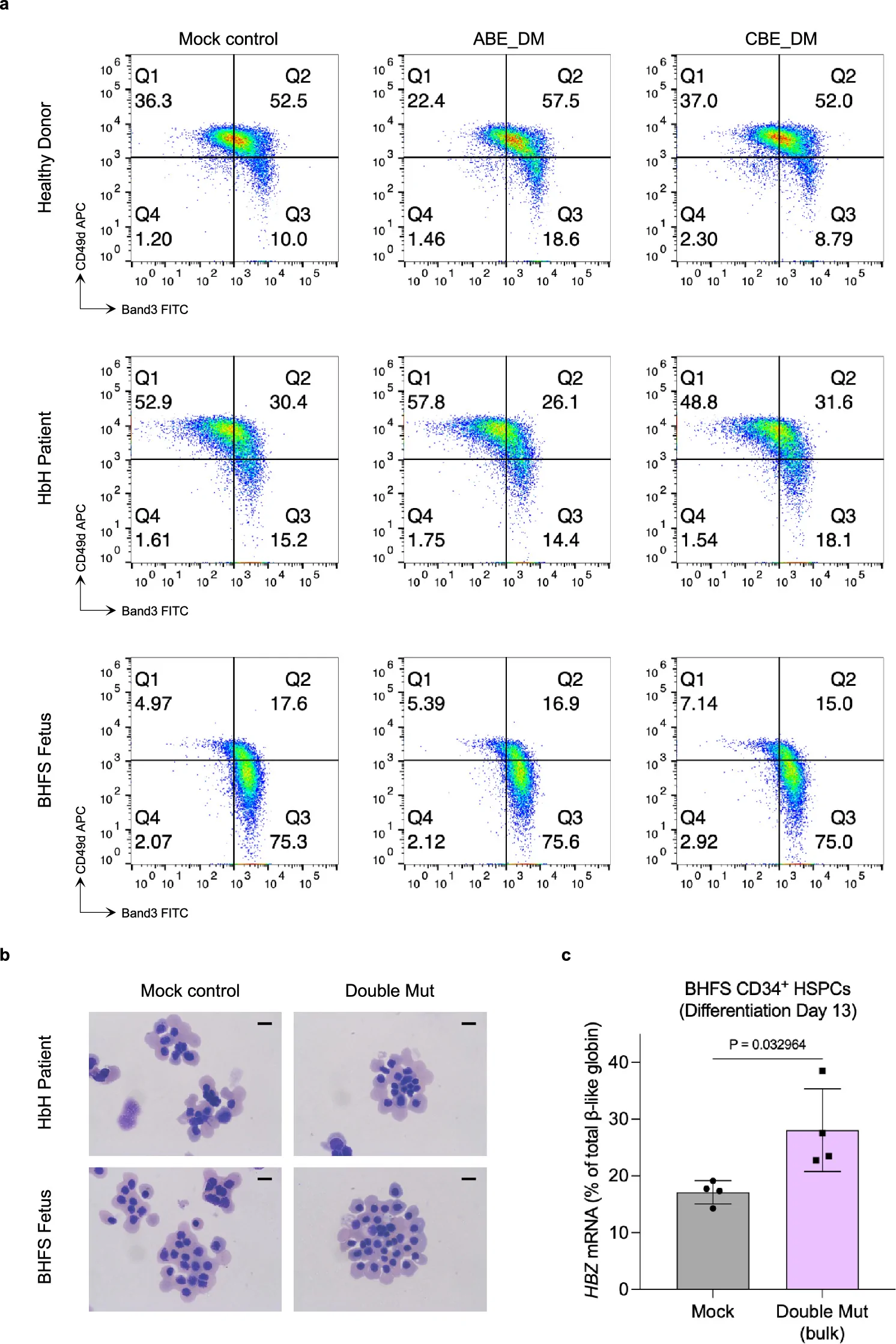
ζ-globin promoter editing in human HSCP-derived primary erythroid cells. **a**, Representative flow cytometry analysis of HSPC-derived erythroid cells from the healthy donor, HbH patient and BHFS fetus, on day 13 of differentiation. **b**, Representative cytospins of HSPC-derived erythroid cells on day 17 of differentiation, stained using a modified Wright’s procedure, scale bar = 10 μm. **c**, Mutation of both repressive motifs increases *HBZ* mRNA in HSPC-derived erythroid cells from a 21-week BHFS fetus (4 independent experiments). Data are presented as mean ± s.d. Exact *P* values are shown in the figure; statistical significance was assessed using a paired two-tailed t-test.

To evaluate the therapeutic potential of this editing strategy in human primary erythroid cells, we edited both repressive elements in CD34^+^ HSPCs from healthy donors, patients with HbH disease (--^SEA^/-α) and a 21-week fetus with BHFS (--^SEA^/--^SEA^). We used either ABE or CBE and achieved editing efficiencies of 25–90% at both sites, with higher efficiencies in fetal than in adult HSPCs (Fig. 6b,e). These edited HSPCs were then differentiated into erythroid cells using our previously described protocol^35^ and monitored by immunophenotyping and modified Wright’s staining. Edited cells undergo normal erythroid differentiation (Extended Data Fig. 6a,b). ζ-globin expression is significantly increased in all three bulk-edited groups, with a more pronounced increase in patient-derived erythroid cells (Fig. 6a and Extended Data Fig. 6c). The percentage of α-like to β-like globin in HbH patients’ samples increases from 24.8% to 48.4% after normalizing to editing efficiency, with ζ-globin accounting for about half of the total α-like globin (Fig. 6c). In erythroid cells derived from edited BHFS fetal HSPCs, ζ-globin expression, after normalizing to editing efficiency, increased from 17.1% to 50.5% (Fig. 6d). The ζ-globin transcriptional activation is also translated to protein expression (Fig. 6f). These data show that this promoter editing strategy reactivates ζ-globin expression in human primary definitive erythroid cells, correcting pathogenic levels of α-like globin (below 25%) to benign levels of ~50% after adjustment for editing efficiency, with the potential of rescuing severe forms of α-thalassemia.

## Discussion

In this study, we delineate a 4.4-kb DNA segment encompassing the embryonic ζ-globin gene that is required for its silencing in definitive erythroid cells and identify two repressive *cis*-regulatory elements within its promoter. Our findings provide a road map for understanding how the ζ-globin gene is silenced in definitive erythropoiesis. We demonstrate that combined disruption of the two identified elements (BCL11A and LRF binding motifs) in the ζ-globin promoter can restore ζ-globin expression to therapeutically meaningful levels in various model systems. Despite weaker dimer interactions and higher oxygen affinity than HbA (α_2_β_2_)^36,37^, there is good evidence to suggest that the resulting hemoglobin molecule Hb Portland 2 (ζ_2_β_2_) may be functional in vivo^7,38^ (Fig. 3e–g). Further analysis of the physiological properties of this hemoglobin in adult life will be required before clinical application. The therapeutic threshold is likely determined not only by the level of ζ-globin expression but also by the biochemical and physiological properties of the resulting hemoglobin molecules. Nevertheless, these results present a potentially viable and rational therapeutic strategy for the treatment of severe α-thalassemia and pave the way for translational clinical trials.

The --^SEA^ allele is the most common deletion causing α^0^-thalassemia, with frequencies reaching up to approximately 7% in certain parts of Southeast Asia, putting up to 1 in 200 pregnancies at risk of BHFS^39,40^. Increasing global migration has led to a greater awareness of BHFS in neonatal units across Europe and North America, broadening its clinical significance beyond traditionally affected regions. Although prenatal screening can prevent late fetal loss and diminish maternal morbidity, BHFS may still present at term in some cases, and survival is now achievable in selected cases through intrauterine transfusions and intensive neonatal care^5^. Nonetheless, all survivors currently depend on lifelong blood transfusions and iron chelation, or on hematopoietic stem cell transplantation when a compatible donor is available. Transfusion needs in BHFS are particularly intensive and surpass those of transfusion-dependent β-thalassemia, yet donor availability remains a limiting factor for transplantation as a generally applicable treatment^38^. Similar to recent developments in therapy for β-thalassemia and sickle cell disease^41–44^, reactivation of a developmentally silenced gene through genome editing of autologous hematopoietic stem cells offers a potential pathway to rescue or even ‘cure’ fetuses and survivors with BHFS and severe HbH disease.

The fetal γ-globin gene can be reactivated to relatively high levels in a wide variety of conditions in adult definitive erythroid cells. In contrast, the embryonic ε-globin and ζ-globin genes seem to be more resistant to reactivation and do not respond to similar circumstances; the reasons are currently unclear. Previous studies have identified the pathway involving the *trans*-acting factors MYB, KLF1, LRF (ZBTB7A) and BCL11A, which have a role in ζ-globin silencing and are associated with localized hypoacetylation of chromatin^10,11,45^. Here using a genetically modified mouse model, we reveal that ζ-globin repression is locally regulated (within a 4.4-kb region). Using saturation mutagenesis, we identify evolutionarily conserved binding sites for BCL11A and LRF within the ζ-globin gene promoter and show that combined mutations of these elements substantially upregulate ζ-globin expression in definitive erythroid cells.

With one exception (Fig. 6d–f), all analyses of BCL11A motif disruption were performed in mouse and human definitive erythroid cells that express the BCL11A-XL isoform, which contains the full zinc finger DNA-binding domain and is associated with developmental globin silencing^46,47^. Consequently, our findings are consistent with interrupting the binding of BCL11A-XL to the ζ-globin promoter. Of interest, mutation of the BCL11A binding site increases ζ-globin expression in early erythroid differentiation, while the effect fades away later. In contrast, mutation of the LRF binding site appears to be effective only in late erythroid differentiation. One possible explanation is the temporally different expression of these two factors—BCL11A is expressed in HSPCs and is increasingly expressed as they differentiate into erythroid cells, whereas LRF is expressed during erythroid differentiation^23^. In HSPCs, when LRF is absent, BCL11A functions as the major ζ-globin repressor, so that disrupting the BCL11A binding site alone activates ζ-globin expression in the early stages of erythropoiesis. Still, ζ-globin becomes resilenced as LRF is switched on later in differentiation. Conversely, as LRF is not expressed in HSPCs, disruption of the LRF binding site alone has no effect during early erythropoiesis. However, it shows a modest derepression later in differentiation when erythroid transcriptional activators such as GATA1 are increasingly expressed and partially counterbalance BCL11A-mediated repression.

Both BCL11A and LRF contribute to the repression of ζ-globin, and LRF seems more effective in maintaining ζ-globin silencing during erythroid differentiation, likely due to the position of their binding sites in the ζ-globin promoter. The proximity of the LRF motif and the CCAAT box (18 bp apart) in the ζ-globin promoter is reminiscent of the role of the BCL11A binding site in the γ-globin promoter, whose occupancy competes with NF-Y binding through steric hindrance to initiate repression^48^. NF-Y binds to the CCAAT box, promoting chromatin accessibility by nucleosome displacement, thereby producing a permissive chromatin conformation for master transcription factors such as GATA1 to bind^49^. Besides, 23 bp upstream of the LRF motif is a GATA1 motif, which may also be blocked by LRF binding. More distally, two additional GATA1 motifs closely surround the BCL11A motif, whose binding may stabilize the repression by hindering GATA1 binding. We speculate that all these elements in the ζ-globin promoter form a tightly orchestrated regulatory module through transcription factor competition—the LRF binding hinders NF-Y binding to maintain a repressive chromatin state, and also acts in concert with BCL11A binding to prevent GATA1 recruitment, thus prohibiting transcriptional initiation of the ζ-globin gene in definitive erythropoiesis.

Existing α-thalassemia models rely predominantly on targeted disruption of α-globin genes and do not reproduce the large *cis*-regulatory rearrangement caused by the --^SEA^ deletion. Our human cell model and mouse model for BHFS precisely recapitulate the --^SEA^/--^SEA^ genotype, providing disease-relevant preclinical models that more closely reflect patient biology. Consistent with observations in --^SEA^ carriers and BHFS fetuses, our BHFS HUDEP-2 cell model shows low-level ζ-globin expression, indicating that this residual activity is an intrinsic property of the locus. However, this low ζ-globin expression is absent in the BHFS mouse model, indicating that some aspects of the reactivation may rely on human-specific mechanisms.

While disrupting BCL11A and LRF consistently increases ζ-globin expression, the effect size of the double-mutation strategy varies across our different model systems. The --^SEA^ deletion markedly increases the effect size of the promoter edits in human primary erythroid cells. The same editing strategy increases ζ-globin expression significantly, albeit minimally in erythroid cells derived from healthy donors (αα/αα), whereas in HbH disease (--^SEA^/-α) and BHFS (--^SEA^/--^SEA^) patient-derived erythroid cells, ζ-globin expression increases markedly by ~23% and ~33%, respectively. With complete biallelic editing, using the strategies described here in patients with HbH disease and BHFS, ζ-globin reactivation would increase the α-like-to-β-like globin ratio to around 0.5, which would likely be sufficient to rescue the phenotype of severe α-thalassemia.

In the future, these levels of ζ-globin expression may be further increased. While BCL11A and LRF appear to be the major repressors of ζ-globin expression in definitive erythroid cells, other elements with less effect may also exist^18^. In addition, there may be embryonic-specific activators that are missing in definitive erythropoiesis. The efficiency of our current editing strategies varies in human primary HSPCs. Given that HSC editing in clinical settings may be lower, follow-up work will be needed to optimize editing strategies and delivery methods to achieve full clinical effectiveness.

It is important to note that the regulatory elements identified in this study are consensus binding motifs for BCL11A and LRF, and direct binding of these factors to the ζ-globin promoter has been shown in other studies^24,25^. In this study, we were unable to show disruption of binding caused by promoter mutations, likely because transcriptional repressors act through a ‘hit and run’ mechanism, making it difficult to catch such interactions using ChIP–seq^50^. It is also possible that BCL11A and LRF can bind their cognate motifs even within the context of ‘closed’ chromatin, but this was not studied further here. However, of interest, we did show that GATA1 newly binds to the ζ-globin promoter when both repressive motifs are mutated. While combinatorial disruption of these repressive elements reactivates ζ-globin expression to levels that are predicted to be clinically useful, the safety of such genome-editing strategies remains to be established in human HSPCs, including rigorous assessment of off-target effects and long-term engraftment. Future work will therefore focus on defining optimized edit combinations, assessing the safety of these approaches in patient-derived HSPCs, and performing preclinical xenotransplantation experiments.

Beyond α-thalassemia, our findings have broader implications for the treatment of genetic diseases in which developmentally silenced paralogous genes remain intact but inactive. By defining a compact, multilayered *cis*-regulatory landscape that enforces embryonic gene silencing, this work reinforces the emerging paradigm, illustrated by γ-globin reactivation in β-thalassemia and sickle cell disease^41–44^, that precise genome editing can be used to unlock latent developmental programs to compensate for deleterious mutations in genes expressed in adults. Such approaches may offer a generalizable route to autologous therapies for severe inherited disorders that currently rely on lifelong supportive care or donor-dependent transplantation.

## Methods

All research was conducted in accordance with relevant ethical regulations. Human studies were approved by the Research Ethics Committee at the University of Oxford (ethics reference: 13/WA/0371), and written informed consent was obtained from all participants or their legally authorized representatives. Animal experiments were approved by the UK Home Office under project license PP2335236 and performed in accordance with guidelines from the Research Ethics Committee at the University of Oxford.

### Mice

All mouse strains in this study were maintained on a C57BL/6J genetic background. WT C57BL/6J mice were obtained from Charles River Laboratories or bred in-house. Mice were housed in individually ventilated cages under specific pathogen-free conditions on a 12-h light/12-h dark cycle, at an ambient temperature of 20–24 °C and relative humidity of 45–65%, with ad libitum access to food and water. The ζ-globin reporter mouse model has been previously reported^10^. The DZIA and SEA mouse models were generated by the Genome Engineering Core at the Centre for Human Genetics, University of Oxford. The ZPE mouse models were generated by the Genome Engineering and Transgenics Facility at the MRC Weatherall Institute of Molecular Medicine, University of Oxford. The guide RNA and donor DNA sequences used to generate these mouse models and primers used for genotyping are listed in Supplementary Table 1. Briefly, the DZIA mouse model was generated by CRISPR-assisted gene targeting in mouse C57BL/6N embryonic stem (ES) cells JM8.F6. Target sites for CRISPR–Cas9 were localized to the extremities of the 4.4 kb *Hba-x* segment. A targeting vector was prepared with 4.9 kb of 5′ and 2.8 kb of 3′ homology flanking a 2.3-kb fragment containing *Hba-alt*. A loxP-flanked neomycin cassette was included to allow selection. ES cells were electroporated with the pX459 CRISPR–Cas9 plasmids and the targeting vector, followed by stable selection. Individual ES cell clones were isolated and screened for homologous recombination. Targeted clones were confirmed by Targeted Locus Amplification (Cergentis). Confirmed ES cells were injected into the pre-implantation blastocysts and resulting chimeras were bred with C57BL/6J mice, followed by Cre deletion of the selection cassette. The SEA mouse model was generated by electroporating C57BL/6J zygotes with sgRNA/Cas9 RNP using Gene Pulser Xcell electroporator (Bio-Rad). The ZPE mouse models were generated by electroporating C57BL/6J WT or SEA heterozygous zygotes with sgRNA/Cas9 RNP and HDR donor DNA oligonucleotide (IDT). After overnight culture, the resulting two-cell embryos were surgically transferred into pseudopregnant recipients and founder mice harboring the expected edits were identified and confirmed by sequencing. Models generated in this study are available from the corresponding authors upon reasonable request.

### Primary mouse erythroid cells

Primitive erythroid cells were isolated from mouse embryonic blood at E10.5–E12.5. Fetal definitive erythroid cells were obtained from mouse fetal livers using an ex vivo culture system^51^. Fetal livers were dissected from E12.5 mouse embryos, and the dissociated cells were expanded for up to 7 days in StemPro-34 (Invitrogen) supplemented with GlutaMAX (Gibco), 100 U ml^−1^ penicillin–streptomycin (Gibco), 50 ng ml^−1^ mouse SCF (PeproTech), 1 U ml^−1^ erythropoietin (OUH Pharmacy) and 0.4 μg ml^−1^ dexamethasone (OUH Pharmacy). HSPCs were isolated by positive selection for CD117 using CD117 microbeads (Miltenyi Biotec) and cultured for at least 24 h to allow erythroid differentiation. Adult definitive erythroid cells were obtained from mouse bone marrow by positive selection for Ter119 using Ter119 microbeads (Miltenyi Biotec).

### G1E-ER4 cell culture

WT G1E-ER4 cells were kindly provided by G.A. Blobel (Children’s Hospital of Philadelphia), originally described in ref. ^22^. G1E-ER4 cells were grown in IMDM media with GlutaMAX (Gibco) with 1.24% monothioglycerol (Sigma-Aldrich), 15% FBS (Gibco), 100 U ml^−1^ penicillin–streptomycin (Gibco), 2 U ml^−1^ erythropoietin (OUH Pharmacy) and 50 ng ml^−1^ mouse SCF (PeproTech) at 37 °C with 5% CO_2_ incubation. Erythroid differentiation was induced by adding 1 mM 4OH-tamoxifen (Sigma-Aldrich) to cultures for 24–48 h and confirmed using flow cytometry and cytospin.

### HUDEP-2 cell culture

WT HUDEP-2 cells were a gift from D. Bauer (Boston Children’s Hospital/Harvard Medical School), originally described in ref. ^26^. HUDEP-2 cells were maintained in the expansion phase using StemSpan SFEM medium (STEMCELL Technologies) supplemented with GlutaMAX (Gibco), 100 U ml^−1^ penicillin–streptomycin (Gibco), 3 U ml^−1^ erythropoietin (OUH Pharmacy), 0.4 μg ml^−1^ dexamethasone (OUH Pharmacy), 50 ng ml^−1^ human SCF (PeproTech) and 2 μg ml^−1^ doxycycline (Sigma-Aldrich) at 37 °C with 5% CO_2_ incubation. To induce differentiation, cells were transferred to IMDM with GlutaMAX (Gibco) supplemented with 3% human AB plasma (NHSBT), 2% FBS (Gibco), 100 U ml^−1^ penicillin–streptomycin (Gibco), 200 μg ml^−1^ holo-transferrin (Sigma-Aldrich), 3 U ml^−1^ heparin (Sigma-Aldrich), 10 μg ml^−1^ insulin (Sigma-Aldrich), 3 U ml^−1^ erythropoietin (OUH Pharmacy), 10 ng ml^−1^ human SCF (PeproTech), 1 ng ml^−1^ human IL-3 (PeproTech) and 2 μg ml^−1^ doxycycline (Sigma-Aldrich). After 5 days, SCF, IL-3 and doxycycline were removed from the media, and cells were then cultured with a high holo-transferrin concentration (500 μg ml^−1^) for further 3–5 days before experiments. Erythroid differentiation was confirmed using flow cytometry and cytospin.

### Primary human CD34^+^ HSPCs

Mononuclear cells were isolated from the peripheral blood of healthy donors, HbH patients and a BHFS fetus using Histopaque (Sigma-Aldrich). HSPCs were enriched by positive selection for CD34 using CD34 microbeads (Miltenyi Biotec) and expanded using StemSpan SFEM II (STEMCELL Technologies) supplemented with 100 ng ml^−1^ human SCF (PeproTech), 100 ng ml^−1^ human TPO (PeproTech), 100 ng ml^−1^ human Flt-3 (PeproTech) and 1 IU ml^−1^ penicillin–streptomycin (Gibco) for 24–48 h at 37 °C and 5% CO_2_ incubation. HSPCs were differentiated into erythroid cells using a published three-phase protocol^35^. Briefly, all phases used a base media of IMDM with GlutaMAX (Gibco) supplemented with 3% human AB plasma (NHSBT), 2% FBS (Gibco), 100 U ml^−1^ penicillin–streptomycin (Gibco), 200 μg ml^−1^ holo-transferrin (Sigma-Aldrich), 3 U ml^−1^ heparin (Sigma-Aldrich) and 10 μg ml^−1^ insulin (Sigma-Aldrich). For phase 1 (days 0–6), cells were cultured in base media supplemented with 3 U ml^−1^ erythropoietin (OUH Pharmacy), 10 ng ml^−1^ human SCF (PeproTech) and 1 ng ml^−1^ human IL-3 (PeproTech). During phase 2 (days 7–10), IL-3 was removed from the phase 1 media. From day 11 onwards, SCF was removed, and additional holo-transferrin was added to a final concentration of 500 μg ml^−1^. Erythroid differentiation was monitored using flow cytometry and cytospin.

### Flow cytometry analysis

Cells were analyzed using the Attune NxT instrument; data were collected using Attune NxT software (v4.2.0) and analyzed using FlowJo (v10.10.0). To monitor erythroid differentiation, cell surface markers were stained using fluorophore-conjugated antibodies. Cells were washed with FACS buffer (PBS with 10% FBS) and resuspended in 100 μl FACS buffer, stained with the following antibodies (1:100 dilution) at 4 °C for 20 min. For mouse cells, PE anti-Ter119 antibody (BD Biosciences, 561071, TER119) and APC anti-CD71 antibody (BD Biosciences, 567258, RI7217) were used. PE anti-CD235a (BD Biosciences, 555570, GA-R2), PerCP-Cy5.5 anti-CD71 (BioLegend, 334114, CY1G4), APC anti-CD49d (BD Biosciences, 561892, 9F10), FITC anti-Band3 (IBGRL, 9439FI, BRIC 6) and APC-Cy7 anti-CD36 (BioLegend, 336213, 5-271) were used for human cells. Cells were washed with FACS buffer, then resuspended in FACS buffer containing 1 ng μl^−1^ of Hoechst 33258 (Invitrogen). Unstained, single-stained and fluorescence minus one controls were prepared for calibration. Intracellular staining of ζ-globin protein was performed using a monoclonal ζ-globin antibody (8E8)^30^. The ζ-globin antibody and a mouse IgG1 isotype control (BD Biosciences, 554121, MOPC-21) were labeled with Alexa Fluor 647 using a Mouse IgG Labeling Kit (Zenon) following the manufacturer’s instructions. Cells were stained using LIVE/DEAD Fixable Aqua Dead Cell Stain Kit (Invitrogen) before being fixed with 3.7% formaldehyde. Fixed cells were then permeabilized using 0.5% Triton X-100 and stained with 1 μg Alexa Fluor 647-labeled ζ-globin antibody or IgG1 isotype control.

### Genome editing

HUDEP-2 cells, primary HSPCs from mice (CD117^+^) and humans (CD34^+^) were transfected using the P3 Primary Cell 4D-Nucleofector kit (Lonza) with either Cas9-sgRNA RNP or base editor mRNA-sgRNA. G1E-ER4 cells were transfected using the SF Cell Line 4D-Nucleofector kit (Lonza). Cas9-sgRNA RNP and base editor mRNA-sgRNA complexes were formed by incubating 1.6 μg of chemically modified synthetic sgRNAs (Synthego) with 3 μg of HiFi Cas9 protein (IDT) or base editor mRNA for 20 min at room temperature. sgRNAs used in the CRISPR screen are listed in Supplementary Table 2; base editor–sgRNA combinations are listed in Supplementary Table 3. Base editor mRNA was produced by in vitro transcription using the HiScibe T7 mRNA (NEB) following the manufacturer’s protocol. For transfection, 0.5–2 × 10^5^ cells were resuspended in 20 μl Nucleofector solution with supplement and mixed with the genome-engineering complex, then electroporated using the Lonza 4D-Nucleofector X Unit (program DZ100 was used for HUDEP-2 cells and human primary HSPCs, program DK100 was used for mouse primary HSPCs and program CM138 was used for G1E-ER4 cells). Immediately after electroporation, cells were placed in expansion media for at least 24 h to recover before differentiating into erythroid cells (for primary HSPCs) or sorting into single cells (to generate clonal G1E-ER4 and HUDEP-2 cell lines). Genomic DNA was extracted from edited cells, and PCR amplicons spanning the edited region were analyzed by Sanger sequencing. Editing efficiencies were quantified using Inference of CRISPR Edits (Synthego), a computational tool that compares edited and control chromatograms to infer frequencies and editing outcomes. Engineered cell lines generated in this study are available from the corresponding authors upon reasonable request.

### Gene expression quantification

RNA was extracted from primary or cultured cells using the Direct-zol Miniprep Kit (Zymo Research) and converted to cDNA using the High-Capacity cDNA Transcription Kit (Applied Biosystems) according to the manufacturer’s instructions. Predesigned TaqMan probes (Applied Biosystems; Supplementary Table 4) and TaqMan Universal PCR Master Mix (Applied Biosystems) were used in qPCR assays to quantify mRNA. Custom primers (synthesized by Merck; Supplementary Table 5) and Luna Universal qPCR Master Mix (NEB) were used in qPCR assays to quantify nascent RNA. qPCR reactions were carried out in 20 µl in technical triplicate on the QuantStudio 3 Real-Time PCR System (Applied Biosystems). RPS18 was used as a reference gene; the ∆Ct method was used for normalized quantification. For bulk-edited cells, normalization was performed by dividing observed phenotypic effects by the fraction of edited alleles caused by the corresponding sgRNA to estimate the effect size in fully edited cells.

### Hemoglobin analysis

Lysates from peripheral blood and differentiated HUDEP-2 cells (day 10) were analyzed by isoelectric focusing (RESOLVE, PerkinElmer) according to the manufacturer’s instructions, running for 55 min at 1,500 V at 14 °C. Lysates from differentiated HUDEP-2 cells (day 10) were analyzed by HPLC (John Radcliffe Hospital) under conditions that detect fast-eluting proteins.

### ATAC–seq

ATAC–seq was performed following a published protocol^52^ using the Illumina Tagment DNA enzyme and buffer kit. A total of 10,000–50,000 cells were used per biological replicate. Cells were lysed in 50 μl of cold lysis buffer (10 mM Tris–HCl, pH 7.4, 10 mM NaCl, 3 mM MgCl_2_, 0.1% IGEPAL CA-630) and spun down at 4 °C for 10 min at 300*g*. Nuclei were then resuspended in 50 μl tagment reaction mix (25 μl buffer, 2.5 μl enzyme and 22.5 μl water) and incubated for 30 min at 37 °C. Transposed fragments were purified using a Qiagen MinElute Kit and then amplified and indexed with Illumina Nextera index primers by PCR.

### ChIPmentation

ChIPmentation experiments were performed as previously described^53^ with modifications. On day 8 of erythroid differentiation, 1–10 × 10^5^ HUDEP-2 cells were fixed with 2 mM disuccinimidyl glutarate for 30 min, followed by 1% formaldehyde for 10 min, then quenched with 125 mM glycine. Fixed cell pellets were washed with PBS, then lysed in cold lysis buffer (50 mM Tris–HCl, pH 8, 10 mM EDTA and 0.5% SDS) for 30 min on ice. The lysate was sonicated with the Covaris ME220 Focused-ultrasonicator to shear the DNA into ~200 to 300 bp fragments. The sonicate was neutralized with 1% Triton X-100 and incubated overnight with Dynabeads Protein A and G (Invitrogen) and 5 μg GATA1 antibody (Abcam, ab181544, EPR17362). Chromatin-bound beads were washed thrice using a low-salt, a high-salt and a LiCl-containing wash buffer, followed by on-bead tagmentation of the immunoprecipitated chromatin using Tagment DNA enzyme (Illumina). Samples were washed with 10 mM Tris–HCl and then amplified and indexed with Illumina Nextera index primers by PCR.

### Next-generation sequencing (NGS) and data analysis

NGS libraries were assessed using D1000 ScreenTape Assays (Agilent) and quantified using NEBNext Library Quant Kit for Illumina (NEB) before being sequenced using NextSeq 500 or 550 kit (v2.5; Illumina). High-output kits (75 cycles) were used for all samples except for ATAC–seq samples from the DZIA mouse model, which were sequenced using mid-output kits (300 cycles), allowing longer reads to distinguish *Hba-alt* gene from *Hba-a1*/*2* genes. NGS data quality was verified through FastQC (v0.11.9). ATAC–seq and ChIPmentation data were aligned to mm10 (mouse) and hg38 (human) using Bowtie 2 (v2.4.2; -k 2, -p 4). Output SAM files were converted to BAM files, then sorted and indexed using SAMtools (v1.17; view, sort and index, respectively). Sorted BAM files were then normalized to RPKM and converted to bigwig files using deepTools (v3.5.6). Data were visualized in the UCSC Genome Browser. Published datasets were downloaded from the NCBI Genome Expression Omnibus (GEO) and analyzed as described.

### Statistics and reproducibility

Statistical analyses in Fig. 2d, Fig. 3d, Fig. 4b,d, Fig. 5b,c, Fig. 6a,d, Extended Data Fig. 3b and Extended Data Fig. 6c were performed using GraphPad Prism (v11.0.2), as described in the corresponding figure legends and Methods. The statistical tests used and sample sizes of independent biological replicates in Fig. 2d, Fig. 3d, Fig. 4b,d, Fig. 5b,c, Fig. 6a,d, Extended Data Fig. 3b and Extended Data Fig. 6c are provided in the relevant figure legends. No statistical method was used to predetermine sample size. Sample sizes were chosen based on previous studies, established practice in the field and practical considerations to provide sufficient power for the analyses performed. Data distribution was assumed to be normal, but this was not formally tested. No data were excluded from the analyses unless explicitly stated. Experimental cohorts were selected based on genotype and consequently randomization was not performed. Data collection and analysis were not performed blindly to the experimental conditions. Independent experiments were repeated in Fig. 3f,g as indicated in the figure legends, and representative data are shown where appropriate.

### Reporting summary

Further information on research design is available in the Nature Portfolio Reporting Summary linked to this article.

## Online content

Any methods, additional references, Nature Portfolio reporting summaries, source data, extended data, supplementary information, acknowledgements, peer review information; details of author contributions and competing interests; and statements of data and code availability are available at https://doi.org/10.1038/s41588-026-02770-0.

## Supplementary information

Reporting Summary

Peer Review File

Supplementary Tables Supplementary Table 1: Guide RNA and donor DNA sequences used to generate mouse models. Supplementary Table 2: sgRNAs used in the saturation mutagenesis of the mouse *Hba-x* gene promoter. Supplementary Table 3: sgRNAs and base editors used to introduce mutations to the ζ-globin promoter. Supplementary Table 4: TaqMan probes used to measure the mRNA expression. Supplementary Table 5: Custom primers used to measure the nascent RNA expression.

## Source data

Source Data Figs. 1–6 Statistical source data.

Source Data Fig. 3 Uncropped IEF gel for Fig. 3f.

Source Data Fig. 4 Uncropped IEF gel for Fig. 4f.

## Data Availability

Raw and processed sequencing data generated in this study are available in the NCBI GEO under accessions GSE320586 (ATAC–seq data in mouse erythroid cells) and GSE328559 (ATAC–seq and ChIPmentation data in HUDEP-2 cells). Published datasets analyzed in this study were downloaded from GEO under accessions GSE108434 (Fig. 1a), GSE104676 and GSE103445 (Extended Data Fig. 4e). Source data underlying the main figures are provided. All other data supporting the findings of this study are available from the corresponding authors on request. Source data are provided with this paper.

## References

[1] Flint, J. et al. High frequencies of α-thalassaemia are the result of natural selection by malaria. *Nature* **321**, 744–750 (1986). 10.1038/321744a0 3713863

[2] Taylor, S. M., Parobek, C. M. & Fairhurst, R. M. Haemoglobinopathies and the clinical epidemiology of malaria: a systematic review and meta-analysis. *Lancet Infect. Dis.* **12**, 457–468 (2012). 10.1016/S1473-3099(12)70055-5 PMC3404513 22445352

[3] Chui, D. H., Fucharoen, S. & Chan, V. Hemoglobin H disease: not necessarily a benign disorder. *Blood* **101**, 791–800 (2003). 10.1182/blood-2002-07-1975 12393486

[4] Songdej, D. et al. An international registry of survivors with Hb Bart’s hydrops fetalis syndrome. *Blood* **129**, 1251–1259 (2017). 10.1182/blood-2016-08-697110 PMC5345731 28057638

[5] Schwab, M. E. et al. The impact of in utero transfusions on perinatal outcomes in patients with α thalassemia major: the UCSF registry. *Blood Adv.* **7**, 269–279 (2022). 10.1182/bloodadvances.2022007823 PMC9860434 36306387

[6] Huang, L. et al. Transforming the treatment of α-thalassemia: a single-center retrospective study on hematopoietic stem cell transplantation in transfusion-dependent pediatric patients. *Ann. Hematol.* **105**, 157 (2026). 10.1007/s00277-026-06855-y PMC12957040 41776114

[7] Russell, J. E. & Liebhaber, S. A. Reversal of lethal α- and β-thalassemias in mice by expression of human embryonic globins. *Blood* **92**, 3057–3063 (1998). 9787139

[8] King, A. J. & Higgs, D. R. Potential new approaches to the management of the Hb Bart’s hydrops fetalis syndrome: the most severe form of α-thalassemia. *Hematology Am. Soc. Hematol. Educ. Program* **2018**, 353–360 (2018). 10.1182/asheducation-2018.1.353 PMC6246003 30504332

[9] Weatherall, D. J. & Clegg, J. B. in *The Thalassaemia Syndromes*, 4th edn (Wiley, 2001).

[10] King, A. J. et al. Reactivation of a developmentally silenced embryonic globin gene. *Nat. Commun.* **12**, 4439 (2021). 10.1038/s41467-021-24402-3 PMC8295333 34290235

[11] Viprakasit, V. et al. Mutations in Kruppel-like factor 1 cause transfusion-dependent hemolytic anemia and persistence of embryonic globin gene expression. *Blood* **123**, 1586–1595 (2014). 10.1182/blood-2013-09-526087 24443441

[12] Gudmundsdottir, B. et al. POGZ is required for silencing mouse embryonic β-like hemoglobin and human fetal hemoglobin expression. *Cell Rep.* **23**, 3236–3248 (2018). 10.1016/j.celrep.2018.05.043 PMC7301966 29898395

[13] Yi, Z. et al. Sox6 directly silences ε globin expression in definitive erythropoiesis. *PLoS Genet.* **2**, e14 (2006). 10.1371/journal.pgen.0020014 PMC1359074 16462943

[14] Hay, D. et al. Genetic dissection of the α-globin super-enhancer in vivo. *Nat. Genet.* **48**, 895–903 (2016). 10.1038/ng.3605 PMC5058437 27376235

[15] Peschle, C. et al. Haemoglobin switching in human embryos: asynchrony of ζ→α and ε→γ-globin switches in primitive and definite erythropoietic lineage. *Nature* **313**, 235–238 (1985). 10.1038/313235a0 2578614

[16] Kingsley, P. D. et al. ‘Maturational’ globin switching in primary primitive erythroid cells. *Blood* **107**, 1665–1672 (2006). 10.1182/blood-2005-08-3097 PMC1895399 16263786

[17] Hua, P. et al. Defining genome architecture at base-pair resolution. *Nature* **595**, 125–129 (2021). 10.1038/s41586-021-03639-4 34108683

[18] Liebhaber, S. A. et al. Developmental silencing of the embryonic ζ-globin gene: concerted action of the promoter and the 3′-flanking region combined with stage-specific silencing by the transcribed segment. *Mol. Cell. Biol.* **16**, 2637–2646 (1996). 10.1128/mcb.16.6.2637 PMC231254 8649371

[19] Russell, J. E. et al. Sequence divergence in the 3′ untranslated regions of human ζ- and α-globin mRNAs mediates a difference in their stabilities and contributes to efficient α-to-ζ gene development switching. *Mol. Cell. Biol.* **18**, 2173–2183 (1998). 10.1128/mcb.18.4.2173 PMC121457 9528789

[20] Cai, W. et al. Enhancer dependence of cell-type-specific gene expression increases with developmental age. *Proc. Natl Acad. Sci. USA* **117**, 21450–21458 (2020). 10.1073/pnas.2008672117 PMC7474592 32817427

[21] Sabath, D. E. et al. Analysis of the human ζ-globin gene promoter in transgenic mice. *Blood* **82**, 2899–2905 (1993). 8219238

[22] Weiss, M. J., Yu, C. & Orkin, S. H. Erythroid-cell-specific properties of transcription factor GATA-1 revealed by phenotypic rescue of a gene-targeted cell line. *Mol. Cell. Biol.* **17**, 1642–1651 (1997). 10.1128/mcb.17.3.1642 PMC231889 9032291

[23] Masuda, T. et al. Transcription factors LRF and BCL11A independently repress expression of fetal hemoglobin. *Science* **351**, 285–289 (2016). 10.1126/science.aad3312 PMC4778394 26816381

[24] Martyn, G. E. et al. Natural regulatory mutations elevate the fetal globin gene via disruption of BCL11A or ZBTB7A binding. *Nat. Genet.* **50**, 498–503 (2018). 10.1038/s41588-018-0085-0 29610478

[25] Liu, N. et al. Direct promoter repression by BCL11A controls the fetal to adult hemoglobin switch. *Cell* **173**, 430–442 (2018). 10.1016/j.cell.2018.03.016 PMC5889339 29606353

[26] Kurita, R. et al. Establishment of immortalized human erythroid progenitor cell lines able to produce enucleated red blood cells. *PLoS ONE* **8**, e59890 (2013). 10.1371/journal.pone.0059890 PMC3606290 23533656

[27] Dierks, P. et al. Three regions upstream from the cap site are required for efficient and accurate transcription of the rabbit β-globin gene in mouse 3T6 cells. *Cell* **32**, 695–706 (1983). 10.1016/0092-8674(83)90055-7 6299573

[28] Mantovani, R. The molecular biology of the CCAAT-binding factor NF-Y. *Gene* **239**, 15–27 (1999). 10.1016/s0378-1119(99)00368-6 10571030

[29] Chui, D. H. et al. Embryonic ζ-globin chains in adults: a marker for α-thalassemia-1 haplotype due to a greater than 17.5-kb deletion. *N. Engl. J. Med.* **314**, 76–79 (1986). 10.1056/NEJM198601093140203 3941693

[30] Luo, H. Y. et al. A novel monoclonal antibody based diagnostic test for α-thalassemia-1 carriers due to the (-SEA/) deletion. *Blood* **72**, 1589–1594 (1988). 3179441

[31] Tang, W. et al. Human embryonic ζ-globin chain expression in deletional α-thalassemias. *Blood* **80**, 517–522 (1992). 1627804

[32] Weatherall, D. J., Clegg, J. B. & Boon, W. H. The haemoglobin constitution of infants with the haemoglobin Bart’s hydrops foetalis syndrome. *Br. J. Haematol.* **18**, 357–367 (1970). 10.1111/j.1365-2141.1970.tb01449.x 5531027

[33] Kutlar, F. et al. The types of hemoglobins and globin chains in hydrops fetalis. *Hemoglobin* **13**, 671–683 (1989). 10.3109/03630268908998844 2634668

[34] Chui, D. H. & Waye, J. S. Hydrops fetalis caused by α-thalassemia: an emerging health care problem. *Blood* **91**, 2213–2222 (1998). 9516118

[35] Scott, C. et al. Recapitulation of erythropoiesis in congenital dyserythropoietic anaemia type I (CDA-I) identifies defects in differentiation and nucleolar abnormalities. *Haematologica* **106**, 2960–2970 (2021). 10.3324/haematol.2020.260158 PMC8561284 33121234

[36] He, Z. & Russell, J. E. Expression, purification, and characterization of human hemoglobins Gower-1 (ζ_2_ε_2_), Gower-2 (α_2_ε_2_), and Portland-2 (ζ_2_β_2_) assembled in complex transgenic-knockout mice. *Blood* **97**, 1099–1105 (2001). 10.1182/blood.v97.4.1099 11159543

[37] Manning, L. R. et al. Human embryonic, fetal, and adult hemoglobins have different subunit interface strengths. Correlation with lifespan in the red cell. *Protein Sci.* **16**, 1641–1658 (2007). 10.1110/ps.072891007 PMC2203358 17656582

[38] Amid, A. et al. Hemoglobin Bart’s hydrops fetalis: charting the past and envisioning the future. *Blood* **144**, 822–833 (2024). 10.1182/blood.2023023692 38457773

[39] Lemmens-Zygulska, M. et al. Prevalence of α-thalassemias in northern Thailand. *Hum. Genet.* **98**, 345–347 (1996). 10.1007/s004390050220 8707307

[40] Shang, X. et al. Rapid targeted next-generation sequencing platform for molecular screening and clinical genotyping in subjects with hemoglobinopathies. *EBioMedicine* **23**, 150–159 (2017). 10.1016/j.ebiom.2017.08.015 PMC5605365 28865746

[41] Sankaran, V. G. & Orkin, S. H. The switch from fetal to adult hemoglobin. *Cold Spring Harb. Perspect. Med.* **3**, a011643 (2013). 10.1101/cshperspect.a011643 PMC3530042 23209159

[42] Orkin, S. H. The fetal-to-adult hemoglobin switch—mechanism and therapy. *N. Engl. J. Med.* **392**, 2135–2149 (2025). 10.1056/NEJMra2405260 40466067

[43] Frangoul, H. et al. CRISPR–Cas9 gene editing for sickle cell disease and β-thalassemia. *N. Engl. J. Med.* **384**, 252–260 (2021). 10.1056/NEJMoa2031054 33283989

[44] Locatelli, F. et al. Exagamglogene autotemcel for transfusion-dependent β-thalassemia. *N. Engl. J. Med.* **390**, 1663–1676 (2024). 10.1056/NEJMoa2309673 38657265

[45] Sankaran, V. G. et al. MicroRNA-15a and -16-1 act via MYB to elevate fetal hemoglobin expression in human trisomy 13. *Proc. Natl Acad. Sci. USA* **108**, 1519–1524 (2011). 10.1073/pnas.1018384108 PMC3029749 21205891

[46] Sankaran, V. G. et al. Human fetal hemoglobin expression is regulated by the developmental stage-specific repressor BCL11A. *Science* **322**, 1839–1842 (2008). 10.1126/science.1165409 19056937

[47] Sankaran, V. G. et al. Developmental and species-divergent globin switching are driven by BCL11A. *Nature* **460**, 1093–1097 (2009). 10.1038/nature08243 PMC3749913 19657335

[48] Liu, N. et al. Transcription factor competition at the γ-globin promoters controls hemoglobin switching. *Nat. Genet.* **53**, 511–520 (2021). 10.1038/s41588-021-00798-y PMC8038971 33649594

[49] Oldfield, A. J. et al. Histone-fold domain protein NF-Y promotes chromatin accessibility for cell type-specific master transcription factors. *Mol. Cell* **55**, 708–722 (2014). 10.1016/j.molcel.2014.07.005 PMC4157648 25132174

[50] Shah, M. et al. Hit and run transcriptional repressors are difficult to catch in the act. *Bioessays* **41**, e1900041 (2019). 10.1002/bies.201900041 31245868

[51] Drissen, R. et al. The erythroid phenotype of EKLF-null mice: defects in hemoglobin metabolism and membrane stability. *Mol. Cell. Biol.* **25**, 5205–5214 (2005). 10.1128/MCB.25.12.5205-5214.2005 PMC1140573 15923635

[52] Buenrostro, J. D. et al. Transposition of native chromatin for fast and sensitive epigenomic profiling of open chromatin, DNA-binding proteins and nucleosome position. *Nat. Methods* **10**, 1213–1218 (2013). 10.1038/nmeth.2688 PMC3959825 24097267

[53] Schmidl, C. et al. ChIPmentation: fast, robust, low-input ChIP–seq for histones and transcription factors. *Nat. Methods* **12**, 963–965 (2015). 10.1038/nmeth.3542 PMC4589892 26280331

