# Supplementary material for: Reactivation of the embryonic ζ-globin gene ameliorates severe forms of α-thalassemia
Source: Nat Genet. 2026 Oct 1;58(10):2619–29. doi: 10.1038/s41588-026-02770-0 (PMC13649559; doi:10.1038/s41588-026-02770-0)
Supplement: Supplementary file 1 [file 41588_2026_2770_MOESM1_ESM.pdf]

nature portfolio

Corresponding author(s): Douglas Higgs, Mira Kassouf

Last updated by author(s): Jul 24, 2026

Reporting Summary

Nature Portfolio wishes to improve the reproducibility of the work that we publish. This form provides structure for consistency and transparency in reporting. For further information on Nature Portfolio policies, see our Editorial Policies and the Editorial Policy Checklist.

Statistics

For all statistical analyses, confirm that the following items are present in the figure legend, table legend, main text, or Methods section.

n/a	Confirmed	
☐	☒	The exact sample size (*n*) for each experimental group/condition, given as a discrete number and unit of measurement
☐	☒	A statement on whether measurements were taken from distinct samples or whether the same sample was measured repeatedly
☐	☒	The statistical test(s) used AND whether they are one- or two-sided<br>*Only common tests should be described solely by name; describe more complex techniques in the Methods section.*
☐	☒	A description of all covariates tested
☐	☒	A description of any assumptions or corrections, such as tests of normality and adjustment for multiple comparisons
☐	☒	A full description of the statistical parameters including central tendency (e.g. means) or other basic estimates (e.g. regression coefficient) AND variation (e.g. standard deviation) or associated estimates of uncertainty (e.g. confidence intervals)
☐	☒	For null hypothesis testing, the test statistic (e.g. *F*, *t*, *r*) with confidence intervals, effect sizes, degrees of freedom and *P* value noted<br>*Give P values as exact values whenever suitable.*
☒	☐	For Bayesian analysis, information on the choice of priors and Markov chain Monte Carlo settings
☒	☐	For hierarchical and complex designs, identification of the appropriate level for tests and full reporting of outcomes
☒	☐	Estimates of effect sizes (e.g. Cohen's *d*, Pearson's *r*), indicating how they were calculated

Our web collection on statistics for biologists contains articles on many of the points above.

Software and code

Policy information about availability of computer code

Data collection	Illumina NextSeq 550 Software v2.2.0 was used to collect the NGS data. Flow cytometry data was collected using Attune NxT software v4.2.0.
Data analysis	NGS data quality was verified via FASTQC (0.11.9). ATAC-seq and ChIPmentation data were aligned to mm10 (mouse) and hg38 (human) using Bowtie2 v2.4.2. Output SAM files were converted to BAM files, then sorted and indexed using Samtools v1.17. Sorted BAM files were then normalised to RPKM and converted to bigwig files using Deeptools v3.5.6. Data were visualised in the UCSC genome browser. Flow cytometry data was analysed using FlowJo v10.10.0. Images of mouse embryos were taken and analysed using Leica Application Suite X v3.7.1.21655. Images of cytospins were taken and analysed using INFINITY ANALYZE v7.0.2.930. Statistical analyses were performed using GraphPad Prism version 11.0.2.

For manuscripts utilizing custom algorithms or software that are central to the research but not yet described in published literature, software must be made available to editors and reviewers. We strongly encourage code deposition in a community repository (e.g. GitHub). See the Nature Portfolio guidelines for submitting code & software for further information.

Data

Policy information about availability of data

All manuscripts must include a data availability statement. This statement should provide the following information, where applicable:

- Accession codes, unique identifiers, or web links for publicly available datasets
- A description of any restrictions on data availability
- For clinical datasets or third party data, please ensure that the statement adheres to our policy

Raw and processed sequencing data generated in this study are available in the NCBI Gene Expression Omnibus (GEO) under accession numbers GSE320586 (ATAC-seq data in murine erythroid cells) and GSE328559 (ATAC-seq and ChIPmentation data in HUDEP-2 cells). Published datasets analysed in this study were downloaded from GEO under accession numbers GSE108434 (Fig. 1a), GSE104676 and GSE103445 (Extended Data Fig. 4e). Source data underlying the main figures are provided. All other data supporting the findings of this study are available from the corresponding authors on request.

Research involving human participants, their data, or biological material

Policy information about studies with human participants or human data. See also policy information about sex, gender (identity/presentation), and sexual orientation and race, ethnicity and racism.

Reporting on sex and gender	Sex and gender were not considered in this study. Findings are expected to apply equally.
Reporting on race, ethnicity, or other socially relevant groupings	Socially relevant groupings were not used in this study.
Population characteristics	The genotypic information of the alpha-globin locus.
Recruitment	Healthy donors were recruited voluntarily. Patients were recruited by the attending physicians from the clinics of the Oxford University Hospitals Trust.
Ethics oversight	Research Ethics Committee at the University of Oxford

Note that full information on the approval of the study protocol must also be provided in the manuscript.

Field-specific reporting

Please select the one below that is the best fit for your research. If you are not sure, read the appropriate sections before making your selection.

☒ Life sciences ☐ Behavioural & social sciences ☐ Ecological, evolutionary & environmental sciences

For a reference copy of the document with all sections, see nature.com/documents/nr-reporting-summary-flat.pdf

Life sciences study design

All studies must disclose on these points even when the disclosure is negative.

Sample size	No statistical method was used to predetermine sample size. Sample sizes were chosen based on previous studies, established practice in the field, and practical considerations to provide sufficient power for the analyses performed.
Data exclusions	No data were excluded from the analyses unless explicitly stated.
Replication	Independent experiments were repeated as indicated, and all attempts at replication were successful, representative data are shown where appropriate.
Randomization	Experimental cohorts were selected based on genotype and consequently randomisation was not performed.
Blinding	Data collection and analysis were not performed blind, as experimental cohorts were selected based on genotype.

Reporting for specific materials, systems and methods

We require information from authors about some types of materials, experimental systems and methods used in many studies. Here, indicate whether each material, system or method listed is relevant to your study. If you are not sure if a list item applies to your research, read the appropriate section before selecting a response.

Materials & experimental systems

n/a	Involved in the study
☐	☒ Antibodies
☐	☒ Eukaryotic cell lines
☒	☐ Palaeontology and archaeology
☐	☒ Animals and other organisms
☒	☐ Clinical data
☒	☐ Dual use research of concern
☒	☐ Plants

Methods

n/a	Involved in the study
☐	☒ ChIP-seq
☐	☒ Flow cytometry
☒	☐ MRI-based neuroimaging

Antibodies

Antibodies used

Antibodies for Flow Cytometry were used in 1:100 dilutions:
PE anti-Ter119 antibody (BD, 561071, TER119)
APC anti-CD71 antibody (BD, 567258, RI7217)
PE anti-CD235a (BD, 555570, GA-R2)
PerCP-Cy5.5 anti-CD71 (BioLegend, 334114, CY1G4)
APC anti-CD49d (BD, 561892, 9F10)
FITC anti-Band3 (IBGRL, 9439FI, BRIC 6)
APC-Cy7 anti-CD36 (BioLegend, 336213, 5-271)
1 ug antibody per test was used for intracellular staining.
mouse IgG1 isotype control (BD, 554121, MOPC-21)
anti-zeta-globin (8E8): As reported in Luo, H.Y., Clarke, B.J., Gauldie, J., Patterson, M., Liao, S.K., and Chui, D.H. (1988). A novel monoclonal antibody based diagnostic test for alpha-thalassemia-1 carriers due to the (--SEA/) deletion. Blood 72, 1589–1594.
5 ug GATA1 antibody (Abcam, ab181544, EPR17362) per test was used for ChIPmentation experiment.

Validation

All the antibodies used in this study were validated by the manufacturers or previous literatures as listed above.

Eukaryotic cell lines

Policy information about cell lines and Sex and Gender in Research

Cell line source(s)

Wild-type G1E-ER4 cells were kindly provided by Gerd A. Blobel (Children's Hospital of Philadelphia), originally described by Weiss et al., 1997.
Wild-type HUDEP-2 cells were a gift from Daniel Bauer (Boston Children's Hospital/Harvard Medical School), originally described by Kurita et al., 2013.

Authentication

Cells were authenticated by aCGH analysis (Moir-Meyer et al, Methods Protoc. 2018) and analysed via cytospins and immunophenotyping during erythroid differentiation.

Mycoplasma contamination

Cells were negative of mycoplasma contamination.

Commonly misidentified lines
(See ICLAC register)

None.

Animals and other research organisms

Policy information about studies involving animals; ARRIVE guidelines recommended for reporting animal research, and Sex and Gender in Research

Laboratory animals

All mouse strains in this study were maintained on a C57BL/6J genetic background. Wild-type C57BL/6J mice were obtained from Charles River Laboratories (UK) or bred in-house. Mice were housed in individually ventilated cages under specific pathogen-free conditions on a 12 h light/12 h dark cycle, at an ambient temperature of 20-24 oC and relative humidity of 45-65%, with ad libitum access to food and water. Both genders aged from embryonic day 10.5 to 6 months were used.

Wild animals

No wild animals were used in this study.

Reporting on sex

Findings apply to both genders.

Field-collected samples

No field collected samples were used in this study.

Ethics oversight

Animal experiments were approved by the UK Home Office under project license PP2335236 and performed in accordance with guidelines from the Research Ethics Committee at the University of Oxford.

Note that full information on the approval of the study protocol must also be provided in the manuscript.

Plants

Seed stocks	*Report on the source of all seed stocks or other plant material used. If applicable, state the seed stock centre and catalogue number. If plant specimens were collected from the field, describe the collection location, date and sampling procedures.*
Novel plant genotypes	*Describe the methods by which all novel plant genotypes were produced. This includes those generated by transgenic approaches, gene editing, chemical/radiation-based mutagenesis and hybridization. For transgenic lines, describe the transformation method, the number of independent lines analyzed and the generation upon which experiments were performed. For gene-edited lines, describe the editor used, the endogenous sequence targeted for editing, the targeting guide RNA sequence (if applicable) and how the editor was applied.*
Authentication	*Describe any authentication procedures for each seed stock used or novel genotype generated. Describe any experiments used to assess the effect of a mutation and, where applicable, how potential secondary effects (e.g. second site T-DNA insertions, mosiacism, off-target gene editing) were examined.*

ChIP-seq

Data deposition

☒ Confirm that both raw and final processed data have been deposited in a public database such as GEO.

☐ Confirm that you have deposited or provided access to graph files (e.g. BED files) for the called peaks.

Data access links<br>*May remain private before publication.*	https://www.ncbi.nlm.nih.gov/geo/query/acc.cgi?acc=GSE328559
Files in database submission	Fastq files and bigwig files.<br>GATA1_WT_HDP2_day8<br>GATA1_DM_HDP2_day8
Genome browser session<br>(e.g. UCSC)	No longer applicable.

Methodology

Replicates	WT and Double Mutant HUDEP-2 cells were differentiated into erythroid cells and fixed with 2 mM DSG and 1% formaldehyde on day 8 of differentiation for ChIPmentation experiments.
Sequencing depth	All experiments contained >20M reads obtained using High-output 75 cycle kits (Illumina), paired end sequencing.
Antibodies	GATA1 antibody (Abcam, ab181544, EPR17362)
Peak calling parameters	ChIPmentation data were not used for genome wide analyses, but only to show local enrichment at the zeta-globin locus.
Data quality	Sequencing quality was verified via FASTQC (0.11.9).
Software	ChIPmentation data were aligned to hg38 (human) using Bowtie2 v2.4.2. Output SAM files were converted to BAM files, then sorted and indexed using Samtools v1.17. Sorted BAM files were then normalised to RPKM and converted to bigwig files using Deeptools v3.5.6. Data were visualised in the UCSC genome browser.

Flow Cytometry

Plots

Confirm that:

☒ The axis labels state the marker and fluorochrome used (e.g. CD4-FITC).

☒ The axis scales are clearly visible. Include numbers along axes only for bottom left plot of group (a 'group' is an analysis of identical markers).

☒ All plots are contour plots with outliers or pseudocolor plots.

☒ A numerical value for number of cells or percentage (with statistics) is provided.

Methodology

Sample preparation	For erythroid differentiation, cells were washed with FACS buffer (PBS with 10% FBS) prior to staining with a panel of fluorophore-conjugated antibodies (1:100 dilution) at 4°C for 20 min. Following antibody staining, the cells were washed and resuspended in FACS buffer containing Hoechst 33258 for live/dead cell analysis.<br>Intracellular staining of ζ-globin protein was performed using a monoclonal ζ-globin antibody (8E8). The ζ-globin antibody and a mouse IgG1 isotype control (BD, 554121) were labelled with Alexa Fluor 647 using a Mouse IgG Labelling Kit (Zenon) following the manufacturer's instructions. Cells were stained using LIVE/DEAD Fixable Aqua Dead Cell Stain Kit (Invitrogen)

before being fixed with 3.7% formaldehyde. Fixed cells were then permeabilised using 0.5% Triton X-100 and stained with 1 ug Alexa Fluor 647 labelled ζ-globin antibody or IgG1 isotype control.

Instrument

ThermoFisher Attune NxT instrument.

Software

Flow cytometry data was collected using Attune NxT software v4.2.0 and analysed using FlowJo v10.10.0.

Cell population abundance

Cell population abundance was determined by flow cytometry analysis. No sorting was conducted.

Gating strategy

Cells were first gated using FSC/SSC for mononuclear cells, followed by gating singlets (FSC-A/FSC-H) and live cells (viability dye). The the populations were then defined based on their cell surface marker expression, as outlined in the Extended Data Figures. The gates were set based on the FMO controls.

☐ Tick this box to confirm that a figure exemplifying the gating strategy is provided in the Supplementary Information.
