# Supplementary material for: Reactivation of the embryonic ζ-globin gene ameliorates severe forms of α-thalassemia
Source: Nat Genet. 2026 Oct 1;58(10):2619–29. doi: 10.1038/s41588-026-02770-0 (PMC13649559; doi:10.1038/s41588-026-02770-0)
Supplement: Supplementary file 2 [file 41588_2026_2770_MOESM2_ESM.pdf]

nature portfolio

Peer Review File

Reactivation of the embryonic ζ-globin gene ameliorates severe forms of α-thalassaemia.

Corresponding Author: Professor Douglas Higgs

Version 0:

Decision Letter:

8th May 2026

Dear Doug,

Your Article entitled "Reactivation of the embryonic ζ-globin gene ameliorates severe forms of α-thalassaemia" has now been seen by 3 referees. You will see from their comments below that while they find your work of interest, some important points are raised, especially by reviewer #3. We are interested in the possibility of publishing your study in Nature Genetics, but would like to consider your response to these concerns in the form of a revised manuscript before we make a final decision on publication.

We therefore invite you to revise your manuscript taking into account all reviewer comments. Please highlight all changes in the manuscript text file. At this stage we will need you to upload a copy of the manuscript in MS Word .docx or similar editable format.

We are committed to providing a fair and constructive peer-review process. Do not hesitate to contact me if there are specific requests from the reviewers that you believe are technically impossible or unlikely to yield a meaningful outcome. I would be happy to discuss the reviewers' comments in detail to help streamline the revision.

When revising your manuscript:

*1) Include a “Response to referees” document detailing, point-by-point, how you addressed each referee comment. If no action was taken to address a point, you must provide a compelling argument. This response will be sent back to the referees along with the revised manuscript.

*2) If you have not done so already please begin to revise your manuscript so that it conforms to our Article format instructions, available
here.
Refer also to any guidelines provided in this letter.

*3) Include a revised version of any required Reporting Summary: https://www.nature.com/documents/nr-reporting-summary.pdf
It will be available to referees (and, potentially, statisticians) to aid in their evaluation if the manuscript goes back for peer review.
A revised checklist is essential for re-review of the paper.

Please be aware of our guidelines on digital image standards.

EXTENDED DATA FIGURES

When re-submitting your manuscript, please ensure that any supplementary figures and tables that are crucial to the manuscript’s conclusions are converted into Extended Data figures and tables to increase visibility of these data. Extended Data figures and tables are online-only (present in the online PDF and full-text HTML versions of the paper), peer-reviewed display items that provide essential background to the article but are not included in the main article due to space constraints. A maximum of ten Extended Data display items (figures and tables) is permitted.

Please use the link below to submit your revised manuscript and related files:

Link Redacted

Note: This URL links to your confidential home page and associated information about manuscripts you may have submitted, or that you are reviewing for us. If you wish to forward this email to co-authors, please delete the link to your homepage.

Please do not hesitate to contact me if you have any questions or would like to discuss these revisions further.

Nature Genetics is committed to improving transparency in authorship. As part of our efforts in this direction, we are now requesting that all authors identified as ‘corresponding author’ on published papers create and link their Open Researcher and Contributor Identifier (ORCID) with their account on the Manuscript Tracking System (MTS), prior to acceptance. ORCID helps the scientific community achieve unambiguous attribution of all scholarly contributions. You can create and link your ORCID from the home page of the MTS by clicking on ‘Modify my Springer Nature account’. For more information please visit please visit www.springernature.com/orcid.

We look forward to seeing the revised manuscript and thank you for the opportunity to review your work.

Sincerely,

Tiago

Tiago Faial, PhD
Chief Editor
Nature Genetics
https://orcid.org/0000-0003-0864-1200

Reviewers' Comments:

Reviewer #1 (Remarks to the Author):

Reactivating the embryonic ζ-globin gene in patients with α-thalassemia is a promising therapeutic strategy. In this study, the authors identified BCL11A and LRF motifs in the ζ-globin regulatory region that contribute to silencing its expression during fetal and adult stages. Disruption of these two motifs reactivated ζ-globin in both mouse models and patient-derived cells. This is an interesting study that provides proof-of-concept for future therapies and general mechanistic insights into developmental gene regulation. Points to address include:

Major

1. BCL11A isoform expression during fetal development: Previous studies have shown that the BCL11A-XL isoform is expressed in adult but not fetal erythroid cells to repress HBG during human erythropoiesis. In that case, how do the shorter isoforms (BCL11A-S or BCL11A-L), which lack the full zinc finger DNA binding domain, repress HBZ during fetal erythropoiesis? Is the BCL11A-XL isoform expressed during mouse fetal erythropoiesis at the time of Hba-x silencing? The authors should reconcile their data with the patterns of BCL11A isoform expression during mouse and human ontogeny.

2. Phenotype of Hba-x-rescued mice: The abstract indicates that deleting the LRF and BCL11A motifs rescues the otherwise prenatal lethal condition of the homozygous SEA mouse until late in development. Presumably, this means that the rescue is not complete. Please provide details.

3. Figure 5: Is it possible to analyze for 4 tetramer in the differentiated HUDEP models, either by HPLC or staining differentiated cells for HbH inclusions? Does reactivation of HBZ reduce 4 tetramer levels?

4. Comparison between single- and double-motif disruption: The authors compare the effects of single-motif disruption and double-motif disruption. In Fig. 2d, the double mutation appears to induce higher Hba-x expression. However, the single mutations were generated using Cas9, whereas the double mutation was generated using CBE. A more appropriate design would be to compare the effects of CBE-mediated single-motif mutations with those of double-motif mutations. In addition, please note whether the three datapoints shown for the double mutant represent three independent clones or replicates from one clone. At a minimum, these issues should be explained as caveats for interpreting the data.

5. Normalization for editing efficiencies: The authors normalized several readouts to editing efficiency, including mVenus+ cells (%) (Fig. 2b), Hba-x expression (Fig. 2c), the percentage of α-like to β-like globin (Fig. 6c), and HBZ expression (Fig. 6d). Extended Data Fig. 2c and 2d show relatively low editing efficiencies for sg39/40/41 (around 20%). Of course,

normalization will amplify the apparent effects of low-efficiency gRNAs. The authors should explain how normalization was performed and provide normalized and non-normalized data.

6. Interpretation of isoelectric focusing results (Fig 4f). Control and manipulated HUDEP2 cells appear to express higher levels of HbF than reported previously. Please clarify and consider validating the result with an orthogonal assay such as HPLC. Could the starting point for the experiment be a clone that expresses an unusually high HbF level?

Minor

1. Abstract: The statement, "α-thalassaemia alone affects hundreds of millions of individuals", is potentially misleading. People are minimally affected by a genetic variant that causes no symptoms (except for genetic implications to offspring). More realistically, hundreds of thousands of people are affected by symptomatic α-thalassemia.

2. Explain "chromatogram analysis by ICE" in the method section.

3. Explain genotyping strategy for distinguishing DZIA and WT alleles in Figure 1a.

4. Use consistent terms for SEA hom, BHFS, and BHFS (SEA hom) in Fig. 3d, 3e, and 3g.

5. The authors mention that "Previous studies suggest that the ζ-globin gene is repressed by sequences within its promoter, gene body, and flanking regions". Can they discuss how sequences in the gene body and flanking region (presumably 3' UTR) might repress the ζ-globin gene? Does the presence of these sequences in double-mutant erythroblasts account for failure to fully de-repress HBZ after mutating the LRF and BCL11A motifs?

6. Autonomous vs. enhancer-dependent regulation of HBZ/Hba-x: The authors have investigated this issue previously. Can results from the current study support current models? For example, if HBZ/Hba-x is regulated fully or partly through interactions with the HS40/ HS26 enhancer, then reactivation would reduce the absolute level of beta globin expression. Can this issue be investigated and/or discussed in relation to the current study?

Reviewer #2 (Remarks to the Author):

This study asks whether reactivating the embryonic ζ-globin gene (HBZ) can compensate for missing α-globin and treat severe α-thalassaemia, including HbH disease and Hb Bart's Hydrops Fetalis Syndrome (BHFS). This is clinically important because the only long-term curative option for these patients is haematopoietic stem cell transplantation, which is limited by donor availability and carries risks such as graft-versus-host disease and transplant-related mortality.
The key idea is that although ζ-globin is normally silenced after early embryogenesis, it can form functional haemoglobin (Hb Portland) and may substitute for α-globin if reactivated. The human α-globin locus contains one embryonic ζ-globin gene (HBZ) and two fetal/adult α-globin genes (HBA1 and HBA2). Using ATAC-seq and H3K27ac ChIP-seq, the authors show that ζ-globin lies in a closed, repressive chromatin region and narrow this silenced domain to a 4.4 kb segment around the gene. They then use CRISPR screening to identify two repressive promoter elements, with binding sites for BCL11A and LRF (ZBTB7A). Disrupting both sites leads to synergistic ζ-globin reactivation.
This strategy was tested in several models, including a BHFS-like mouse model, HUDEP-2 cells, and primary human CD34+ cells from patients with HbH disease and BHFS. In primary patient-derived cells, ζ-globin reactivation restores α-like globin to approximately therapeutic levels (~50% after normalisation). Overall, the authors conclude that ζ-globin silencing is controlled by specific promoter elements and that editing these elements may provide a gene-editing approach for severe α-thalassaemia.

The study is novel, biologically elegant, and highly translational. It also suggests a broader therapeutic principle: reactivating developmentally silenced paralogues may be a useful strategy for other genetic diseases.

Minor comments:
1. The authors state that the proximity to α-globin enhancers may explain why a single Hba-alt gene produces about the same output as two endogenous α-globin genes. This point could be explained in more detail.
2. Why was exon 3 chosen for replacement with mVenus?
3. The BCL11A disruption had only a modest effect (~4% of total α-like globin). Was this because only sg33 was chosen? Would targeting a broader region, such as sg32–33, improve reactivation?
4. Have the authors assessed potential off-target genomic events of editing the ζ-globin promoter?
5. Editing efficiency varied widely (25–90%). This should be discussed, especially given that HSC editing in clinical settings may be lower.
6. The manuscript should also discuss lack of oral drugs for treating HbH disease until recently when mitapivat, a pyruvate kinase activator, that has recently been approved for treating both alpha and beta thalassaemia. Gene editing approach should be discussed in this context.

Reviewer #3 (Remarks to the Author):

A. In this manuscript Liu et al. map cis-regulatory elements that silence embryonic alpha-globin gene (zeta) transcription with the aim of reactivating zeta-gene expression to ameliorate alpha-thalassemia eventually in a clinical setting. As a first step, they employ engineered mice to localize silencing elements to a 4.4 kb region containing the zeta-gene. Functional mapping with a Venus zeta-reporter mouse identified BCL11A and LRF binding motifs. Inactivation of both achieves substantial reactivation (~26%). To test the consequences of reactivation in a preclinical model, they proceed to use mice with a deletion that replicates the naturally occurring HbBarts allele (BHFS mice). The BHFS mice mimic the human phenotype. ZPE (zeta promoter edited) mice, in which both distal BCL11A and LRF motif are mutated, appear near normal at E17.5 (with expression of HbPortland II (zeta2beta2), suggesting "translational potential" in severe a-thalassemia. To pursue human relevance of their findings, they generate a HUDEP cell model, and examine consequences of mutation of the binding motifs in CD34 cells. In the human context, reactivation of zeta-globin appeared somewhat better than in mouse, which they interpret as reassuring for translation to patients. In essence, the manuscript has two aspects: 1) mapping of the sequences that silence zeta-globin gene expression in adult cells; and 2) preclinical studies for translation to severe alpha-thalassemia.
B. The studies in this manuscript are well executed. Principal novelty relates to the engineering and use of mouse alleles to reveal in vivo consequences of mutations. The mapping of the critical promoter regulatory sequences, culminating in identification of BCL11A and LRF motifs, is conventional. Prior studies (eg ref 23) pointed to those sequences. It is somewhat disappointing that the "missing" portion of silencing (ie that accounting for substantial residual expression in the context of combined BCL11A and LRF mutation) remains obscure. In terms of significance, the underlying hypothesis that zeta-globin reactivation might be a therapeutic approach to treatment of severe alpha-thalassemia rests primarily on the documented success of gamma-globin reactivation to provide functional cures of sickle cell disease and beta-thalassemia. Surprisingly, this logical foundation for the present studies is not mentioned in the text. This is a glaring omission. Prior genetic and family studies provided firm evidence that reactivation of gamma-globin expression would be of major therapeutic benefit. The same cannot be said regarding reactivation of zeta-globin expression (as noted in part F below). In the opinion of this reviewer, the authors are glib about the therapeutic benefits that might accrue from zeta-globin reactivation.
C. On the whole, the data and execution are excellent. The presentation is solid except for the issues that relate to translaltion of the findings (see below).
D. Statistical evaluation of results is generally solid with one exception: In the section "Identification of represive....mouse zeta-globin gene promoter", they describe the effect of combined BCL11A and LRF motif disruption as "syngergistic", where individual mutation leads to 4 and 18% zeta-expression and the combined mutation 26% (which is not far from 22%, the sum of individual mutations).
E. The conclusions with respect to the consequences of mutation of the BCL11A and LRF motifs in the zeta-promoter are solid, although perhaps not very surprising.
F. Suggested improvements:
Specific points:
1. Fig 1a: the arrow from Hbq1b to Hba-x may be confusing. It is not explained what it is meant to depict (minor comment)
2. The silencing elements are contained within the 4.4 kb region. The presumption is that they are in the promoter. In the experiments in which a Venus reporter is substituted for a portion of the zeta gene, can one exclude the possibility that some silencing sequences might be within the exon 3 deletion?
3. In the ZPE mouse model experiments, it is shown that augmented zeta expression improves the phenotype at E17.5. It is unexplained why liveborn mice (and perhaps older mice) were not studied. Given concerns regarding the in vivo functionality of Portland II, this experiment would seem important to provide.
4. In lines 226-230, the authors note that the proximal BCL11A site overlaps the CCAAT box, precluding mutation of that site. However, perhaps the proximal site is part of the "missing" silencing? Would it be possible to engineer a mutation of the BCL11A site that preserves the CCAAT box? The authors should consider this possibility and perhaps perform an experiment to address this issue directly.
5. In lines 384/5, it is suggested that the inability to show disruption of binding of BCL11A
and LRF is due to their "hit and run" action. This is a speculation. It is equally possible that the problem related to the closed nature of the target sites and inaccessibility (minor point).
6. As noted above, in order to place this work in the proper context, the authors should cite parallel work on the reactivation of gamma-globin as a strategy to treat the beta-hemoglobin disorders.
7. In this reviewer's opinion, the authors should be more critical in their assessment of the potential of zeta-globin reactivation to treat severe alpha-thalassemias. The strongest evidence in favor is ref 7 in which transgenic rescue of alpha-gene deleted mice was reported. In the published manuscript there are precious few details regarding the level of expression of the transgene and the hematology of the rescued mice. There are several reasons to suspect that Portland II may not substitute for HbA as well as implied in the current work. For example, as reported in Protein Science 16: 1641, 2007), the dimer strength of HbPortland II is far weaker than that of HbA or F, and the oxygen affinity is much higher. The difference in dimer strength may suggest that Portland II may be less stable. Since there are no data in the human context demonstrating that Portland II may substitute for HbA in vivo, I would suggest that the authors be more circumspect regarding the clinical application of the approach. The clinical potential of the approach will rest on not only the level at which zeta-globin is reactivated, but likely more importantly on the properties of Portland II and effects on cell lifespan and on oxygen delivery to tissues. These are largely unknown and the authors should acknowledge this.
G. References: Citation of prior work on HbF reactivation should be added. Also, publications on the properties of Portland II (as above) should be included.
H. Clarity and context: no issues, other than those mentioned above.

Version 1:

Decision Letter:

Our ref: NG-A72314R

1st July 2026

Dear Doug,

Thank you for submitting your revised manuscript entitled "Reactivation of the embryonic ζ-globin gene ameliorates severe forms of α-thalassaemia" (NG-A72314R). It has now been seen by the original referees and their comments are below. The reviewers find that the paper has improved in revision, and therefore we'll be happy in principle to publish it in Nature Genetics, pending minor revisions to comply with our editorial and formatting guidelines.

We are now performing detailed checks on your paper and will send you a checklist detailing our editorial and formatting requirements soon. Please do not upload the final materials and make any revisions until you receive this additional information from us.

Thank you again for your interest in Nature Genetics. Please do not hesitate to contact me if you have any questions.

Congratulations!

Sincerely,

Tiago

Tiago Faial, PhD
Chief Editor
Nature Genetics
https://orcid.org/0000-0003-0864-1200

Reviewer #1 (Remarks to the Author):

I am satisfied with the authors' responses to my suggestions. This is an interesting paper that advances the field.

Reviewer #2 (Remarks to the Author):

The authors have addressed our concerns where they could and in places they could not provided an explanation as to why.
“Have the authors assessed potential off-target genomic events of editing the ζ-globin promoter?
We have not assessed the off-target effects of editing the ζ-globin promoter in this study. Because the current study was designed to establish biological proof-of-principle rather than develop a clinical product, comprehensive off-target assessment was considered beyond its scope. In the discussion, we addressed this as our plan, and our future work will focus on assessing the safety of these approaches in patient-derived HSPCs, including off-target effects and long-term engraftment.”

“The manuscript should also discuss lack of oral drugs for treating HbH disease until recently when mitapivat, a pyruvate kinase activator, that has recently been approved for treating both alpha and beta thalassaemia. Gene editing approach should be discussed in this context.
We thank the reviewer for this suggestion and agree that it is important to consider emerging therapeutic approaches for HbH disease, including mitapivat and other pharmacological interventions. A comprehensive comparison of genome editing with alternative therapeutic strategies would substantially broaden the scope of the discussion beyond the primary mechanistic focus of the current study. We have therefore limited our discussion to a brief contextual overview while focusing on the biological insights and proof-of-principle findings presented here.
We appreciate that pharmacological and gene-editing approaches may ultimately prove complementary, with pharmacological therapies targeting ineffective erythropoiesis and red cell metabolism, while gene editing offers the possibility of durable molecular correction.”

Reviewer #3 (Remarks to the Author):

The revision has responded to the critiques of the reviewers. It is a shame that they cannot examine liveborn mice as that

would add to an understanding as to whether the proposed therapeutic approach may be feasible.

Open Access This Peer Review File is licensed under a Creative Commons Attribution 4.0 International License, which permits use, sharing, adaptation, distribution and reproduction in any medium or format, as long as you give appropriate credit to the original author(s) and the source, provide a link to the Creative Commons license, and indicate if changes were made.
In cases where reviewers are anonymous, credit should be given to 'Anonymous Referee' and the source.
The images or other third party material in this Peer Review File are included in the article's Creative Commons license, unless indicated otherwise in a credit line to the material. If material is not included in the article's Creative Commons license and your intended use is not permitted by statutory regulation or exceeds the permitted use, you will need to obtain permission directly from the copyright holder.
To view a copy of this license, visit https://creativecommons.org/licenses/by/4.0/

Reviewers' Comments:

Reviewer #1 (Remarks to the Author):

Reactivating the embryonic ζ-globin gene in patients with α-thalassemia is a promising therapeutic strategy. In this study, the authors identified BCL11A and LRF motifs in the ζ-globin regulatory region that contribute to silencing its expression during fetal and adult stages. Disruption of these two motifs reactivated ζ-globin in both mouse models and patient-derived cells. This is an interesting study that provides proof-of-concept for future therapies and general mechanistic insights into developmental gene regulation. Points to address include:

Major

1. BCL11A isoform expression during fetal development: Previous studies have shown that the BCL11A-XL isoform is expressed in adult but not fetal erythroid cells to repress HBG during human erythropoiesis. In that case, how do the shorter isoforms (BCL11A-S or BCL11A-L), which lack the full zinc finger DNA binding domain, repress HBZ during fetal erythropoiesis?

Is the BCL11A-XL isoform expressed during **mouse** fetal erythropoiesis at the time of Hba-x silencing?

Yes, as the reviewer points out, mouse fetal liver cells do express the long BCL11A-XL isoform (Sankaran et al., Science 2008; Nature 2009) and the Hba-x gene is silenced during mouse fetal erythropoiesis. Mouse fetal definitive erythroblasts behave exactly like adult erythroblasts. The results in this manuscript are therefore entirely consistent with the BCL11A isoform expression in mouse fetal and adult definitive erythropoiesis.

The authors should reconcile their data with the patterns of BCL11A isoform expression during mouse and **human** ontogeny.

The human erythroid cells we used in this study, HUDEP-2 and HSPC-derived erythroid cells, are considered models of adult erythropoiesis that express high levels of the long BCL11A-XL isoform. Our results are therefore consistent with the BCL11A isoform expression in human adult definitive erythropoiesis.

The only experiment using human fetal definitive erythroid cells in the current report is illustrated in Fig. 6d-f, where HSPCs were obtained from a fetus with BHFS. These are highly selected cells in the hydrops fetus that produce small amounts of zeta-globin in the absence of all four alpha-globin genes ($--^{SEA}/--^{SEA}$), thereby allowing the fetus to survive. They are therefore not typical of normal fetal erythroid cells, and their status with respect to BCL11A isoforms is currently unknown. We have now explained this in the figure legend, lines 770-773:

Fig. 6d, Mutation of both repressive motifs increases *HBZ* mRNA in HSPCs-derived erythroid cells from a 21-week BHFS fetus (4 independent experiments). Expression of BCL11A isoforms in these cells is unknown.

Nevertheless, it is clearly shown in Fig. 6d-f that when the BCL11A and LRF binding sites are edited in the BHFS fetal erythroid cells, there is a significant increase in zeta-globin expression. At present, the extent to which the BCL11A motif mutation alone contributes to the observed effects is not known.

The reviewer raises interesting and important mechanistic points for future studies, but this does not detract from the new observations that disrupting the BCL11A and LRF binding sites in the zeta-globin promoter in patient-derived (HbH adult and BHFS fetal) and mouse (adult and fetal) definitive erythroid cells significantly increases zeta-globin expression and can rescue the severe alpha-thalassaemia phenotype.

We have now discussed this point in the manuscript, lines 337-341:

With one exception (Fig. 6d-f), all analyses of BCL11A motif disruption were performed in mouse and human definitive erythroid cells that express the BCL11A-XL isoform, which contains the full zinc finger DNA binding domain and is associated with developmental globin silencing [46, 47]. Consequently, our findings are consistent with interrupting the binding of BCL11A-XL to the ζ-globin promoter.

2. Phenotype of Hba-x-rescued mice: The abstract indicates that deleting the LRF and BCL11A motifs rescues the otherwise prenatal lethal condition of the homozygous SEA mouse until late in development. Presumably, this means that the rescue is not complete. Please provide details.

We agree that our original wording may imply complete rescue beyond late gestation. We have revised the abstract to clarify that our conclusions are based on analysis of embryos close to birth at E17.5. At this stage, Hba-x-rescued embryos appeared morphologically comparable to wild-type littermates and showed marked correction of erythroid abnormalities (Fig. 3g).

However, limitations on our current animal experimentation licence mean that we are not permitted to assess postnatal survival or long-term viability in case the level of zeta-globin reactivation in the mouse model is insufficient for a complete rescue. Allowing potentially severely affected animals to proceed to birth would raise animal welfare concerns on our current licence. Although we are currently unable to assess this, we do anticipate that the BHFS phenotype will be significantly ameliorated postnatally and will provide a potential treatment for this condition.

Proceeding with a plan to take this project from a proof-of-concept to therapeutic development, we are now applying for funding and setting up appropriate collaborations to demonstrate the efficacy of this approach postnatally. For example, animal study plans will include experiments with pre-natal and post-natal blood transfusions to allow 'treated' animals to be born and assess the efficacy and durability of the rescue postnatally and into adulthood. However, this work lies beyond the current study.

3. Figure 5: Is it possible to analyze for β4 tetramer in the differentiated HUDEP models, either by HPLC or staining differentiated cells for HbH inclusions? Does reactivation of HBZ reduce β4 tetramer levels?

We thank the reviewer for this suggestion. We initially performed Brilliant Cresyl Blue (BCB) staining for HbH inclusions, but the results were uninterpretable as HUDEP-2 cells are still nucleated after differentiation, and BCB stains nuclei. To address this point, we have now performed HPLC on differentiated HUDEP-2 cell models under conditions that detect fast-eluting proteins, and there was a reduced early peak indicating reduced Hb H/Bart's (β4/γ4) in double-mutant BHFS HUDEP-2 cells, now added to Extended Fig. 5c and the Main text, lines 271-274:

Importantly, HPLC analysis demonstrated that the double mutation was associated with reduced β-like globin tetramers (Hb H/Bart's) in BHFS HUDEP-2 cells, providing functional evidence that ζ-globin can compensate for α-globin deficiency and ameliorate globin chain imbalance (Extended Data Fig. 5c).

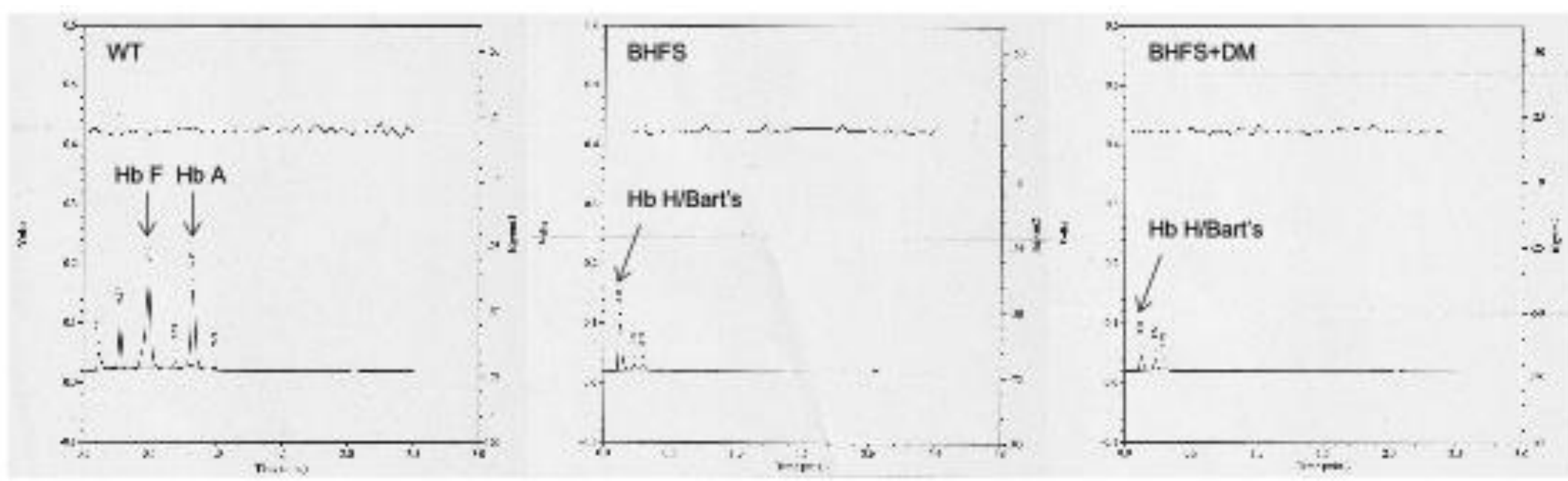

4. Comparison between single- and double-motif disruption: The authors compare the effects of single-motif disruption and double-motif disruption. In Fig. 2d, the double mutation appears to induce higher Hba-x expression. However, the single mutations were generated using Cas9, whereas the double mutation was generated using CBE. A more appropriate design would be to compare the effects of CBE-mediated single-motif mutations with those of double-motif mutations. In addition, please note whether the three datapoints shown for the double mutant represent three independent clones or replicates from one clone. At a minimum, these issues should be explained as caveats for interpreting the data.

We thank the reviewer for this important point. We agree that the comparison between the single-motif and double-motif perturbations should be interpreted cautiously, for the reasons presented by the reviewer. We have now explicitly acknowledged this caveat in the Main text and have tempered our interpretation accordingly, lines 161-165:

The double mutant cells show a higher (~26%) ζ-globin upregulation (Fig. 2d), suggesting a combined effect that is marginally greater than the sum of its parts. It is worth noting that the single- and double-mutant cell lines were generated using different methods and therefore may not be strictly comparable.

The results in Fig. 2d are from independent clones (single mutant), and replicates of one clone (double mutant), and this has now been explained in the figure legend, lines 709-711:

d, Percentage of *Hba-x* mRNA to total α-like globin in clonal G1E-ER4 cell lines (3 independent single mutant clones, 3 independent replicates of a double mutant clone).

We agree that an appropriate comparison would be to compare the effects of CBE-mediated single- and double-mutations. This comparison was performed in HUDEP-2 cells (Fig. 4b, d), in which all models were generated using CBE, and results suggest the two motifs act synergistically.

5. Normalization for editing efficiencies: The authors normalized several readouts to editing efficiency, including mVenus+ cells (%) (Fig. 2b), Hba-x expression (Fig. 2c), the percentage of α-like to β-like globin (Fig. 6c), and HBZ expression (Fig. 6d). Extended Data Fig. 2c and 2d show relatively low editing efficiencies for sg39/40/41 (around 20%). Of course, normalization will amplify the apparent effects of low-efficiency gRNAs. The authors should explain how normalization was performed and provide normalized and non-normalized data.

We agree that normalisation to editing efficiency can amplify apparent effect sizes for lower-efficiency guides and therefore requires careful interpretation. We have now clarified in the Methods how normalisation was performed, lines 519-522:

For bulk-edited cells, normalisation was performed by dividing observed phenotypic effects by the fraction of edited alleles caused by the corresponding sgRNA to estimate the effect size in fully edited cells.

We were also concerned about overestimating the effect size of low-efficiency sgRNAs (sg39/40/41), so we generated clonal cell lines from bulk-edited cells to confirm the actual effect size (Fig. 2d), which is in line with the normalised effect in the bulk population.

To address the reviewer's concern, we now provide normalised data in Fig. 2b, 2c, 6c and 6d, non-normalised data in Extended Data Fig. 2c and 2d (upper graphs), Fig. 6a and Extended Data Fig. 6c. Importantly, the principal conclusions are also evident in the non-normalised datasets.

6. Interpretation of isoelectric focusing results (Fig 4f). Control and manipulated HUDEP2 cells appear to express higher levels of HbF than reported previously. Please clarify and consider validating the result with an orthogonal assay such as HPLC. Could the starting point for the experiment be a clone that expresses an unusually high HbF level?

Yes, we noticed that our parental WT HUDEP-2 cells express higher levels of γ-globin and HbF (confirmed by RNA and HPLC analyses) than previously reported. We speculate that this may result from differences in culture conditions, passage history and clonal variation, which are known to affect globin expression profiles in HUDEP-2 cells.

In this study, all control and edited cells were derived from the same parental WT HUDEP-2 line and analysed in parallel, such that the key comparisons reflect relative differences between matched conditions. Specifically, we use total β-like globin (HBG+HBB) when calculating the relative expression of HBZ.

Minor

1. Abstract: The statement, “α-thalassaemia alone affects hundreds of millions of individuals”, is potentially misleading. People are minimally affected by a genetic variant that causes no symptoms (except for genetic implications to offspring). More realistically, hundreds of thousands of people are affected by symptomatic α-thalassemia.

This sentence has been removed.

2. Explain “chromatogram analysis by ICE” in the method section.

This has now been explained in the Methods, lines 506-509:

Genomic DNA was extracted from edited cells, and PCR amplicons spanning the edited region were analysed by Sanger sequencing. Editing efficiencies were quantified using Inference of CRISPR Edits (ICE; Synthego), a computational tool that compares edited and control chromatograms to infer frequencies and editing outcomes.

3. Explain genotyping strategy for distinguishing DZIA and WT alleles in Figure 1a.

This has now been explained by illustrating the primer pairs used for genotyping in Fig. 1a, and in the figure legend, lines 697-699:

The resulting DZIA allele is illustrated in the diagram below, and genotyping primer pairs used to distinguish the DZIA and WT alleles are indicated by harpoon arrows.

4. Use consistent terms for SEA hom, BHFS, and BHFS (SEA hom) in Fig. 3d, 3e, and 3g.

These terms have been modified. SEA hom indicates the genotype of fetal liver cells in Fig. 3d. BHFS indicates the preclinical mouse model with the SEA hom genotype that develops the BHFS phenotype in Fig. 3e and 3g.

5. The authors mention that "Previous studies suggest that the ζ-globin gene is repressed by sequences within its promoter, gene body, and flanking regions”. Can they discuss how sequences in the gene body and flanking region (presumably 3’ UTR) might repress the ζ-globin gene? Does the presence of these sequences in double-mutant erythroblasts account for failure to fully de-repress HBZ after mutating the LRF and BCL11A motifs?

We thank the reviewer for this insightful comment. Previous studies have suggested that repression of the ζ-globin gene is mediated not only by its proximal promoter, but also by sequences within the gene body and flanking regions (Liebhaber et al., 1996). Although the precise mechanisms remain incompletely defined, these regions may contribute to repression through recruitment of transcriptional corepressors, modulation of chromatin accessibility, effects on transcription elongation, RNA processing/stability, or higher-order chromatin organisation.

We agree that persistence of additional *cis*-regulatory sequences in our double-mutant models might contribute to the incomplete de-repression of ζ-globin. We have now expanded the Discussion to clarify that our data suggest that BCL11A and LRF play a major

role in ζ-globin silencing, and additional regulatory mechanisms may also contribute to full developmental repression of the ζ-globin gene, lines 389-392:

In the future, these levels of ζ-globin expression may be further increased. While BCL11A and LRF appear to be the major repressors of ζ-globin expression in definitive erythroid cells, other elements with less effect may also exist [18]. In addition, there may be embryonic-specific activators that are missing in definitive erythropoiesis.

6. Autonomous vs. enhancer-dependent regulation of HBZ/Hba-x: The authors have investigated this issue previously. Can results from the current study support current models? For example, if HBZ/Hba-x is regulated fully or partly through interactions with the HS40/ HS26 enhancer, then reactivation would reduce the absolute level of beta (alpha?) globin expression. Can this issue be investigated and/or discussed in relation to the current study?

In our previous enhancer knockout mouse models, we have shown that ζ-globin expression is much less reliant on enhancers than α-globin during primitive erythropoiesis. Only about half of its activity in primitive erythroid cells depends on its interaction with the enhancers (Hay et al., 2016; King et al., 2021).

In the current study, we primarily focused on reactivation of ζ-globin rather than on total α-globin output or quantitative enhancer competition. While our data show increased ζ-globin expression following disruption of the LRF and BCL11A motifs, this does not distinguish between autonomous promoter de-repression and enhancer redistribution models.

Notably, in fetal liver cells from the ZPE mouse model, when the ζ-globin gene is reactivated, α-globin expression is slightly reduced (now added to Extended Data Fig. 3b), supporting the hypothesis that ζ-globin activation is partially regulated through interactions with the enhancers. On the other hand, in definitive erythroid cells from the SEA mouse model, deletion of α-globin genes does not result in ζ-globin upregulation (Fig. 3d), suggesting that the ζ-globin gene is actively repressed and cannot be derepressed just via interaction with the enhancers. Overall, the ζ-globin gene seems to exhibit weak competitive activity for the shared enhancer only when its promoter repression is removed, although this was not directly tested in the current study.

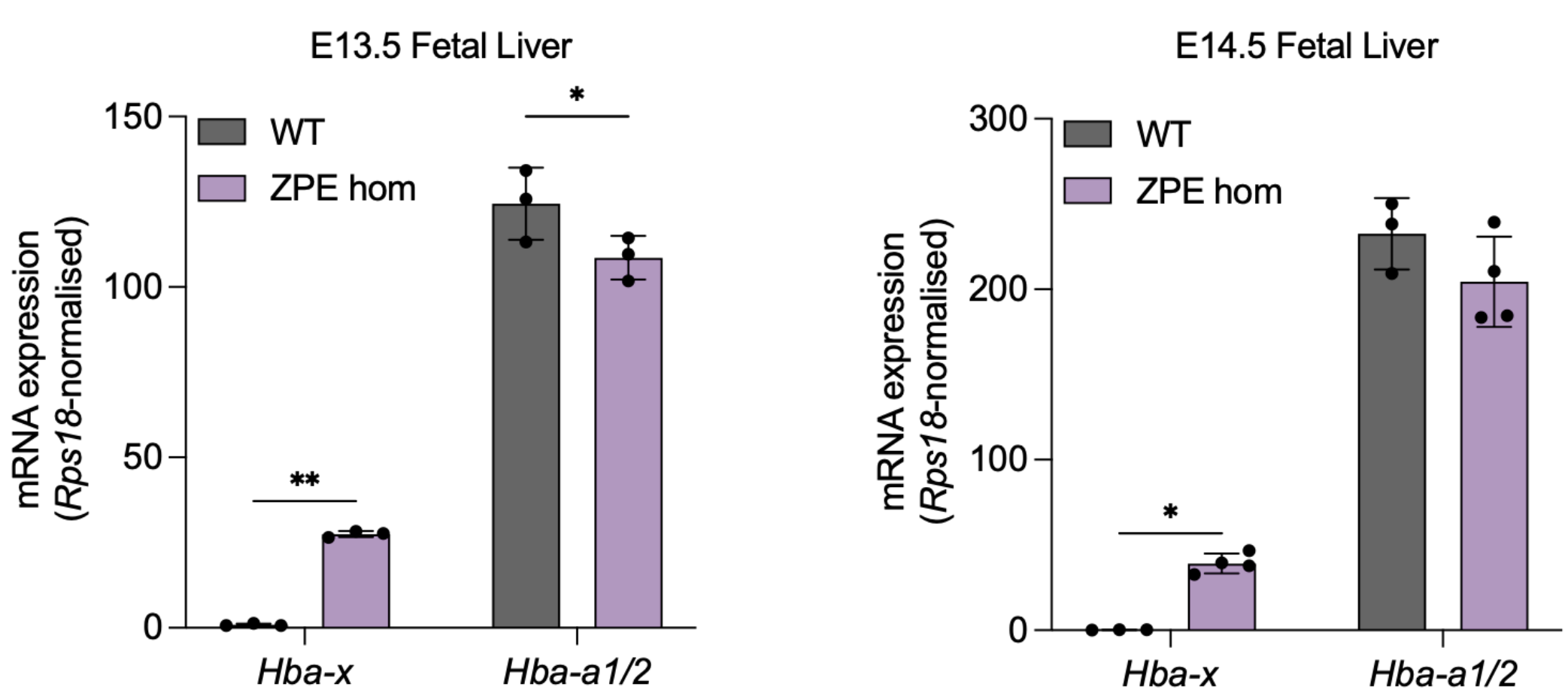

Reviewer #2 (Remarks to the Author):

This study asks whether reactivating the embryonic ζ-globin gene (HBZ) can compensate for missing α-globin and treat severe α-thalassaemia, including HbH disease and Hb Bart's Hydrops Fetalis Syndrome (BHFS). This is clinically important because the only long-term curative option for these patients is haematopoietic stem cell transplantation, which is limited by donor availability and carries risks such as graft-versus-host disease and transplant-related mortality.

The key idea is that although ζ-globin is normally silenced after early embryogenesis, it can form functional haemoglobin (Hb Portland) and may substitute for α-globin if reactivated. The human α-globin locus contains one embryonic ζ-globin gene (HBZ) and two fetal/adult α-globin genes (HBA1 and HBA2). Using ATAC-seq and H3K27ac ChIP-seq, the authors show that ζ-globin lies in a closed, repressive chromatin region and narrow this silenced domain to a 4.4 kb segment around the gene. They then use CRISPR screening to identify two repressive promoter elements, with binding sites for BCL11A and LRF (ZBTB7A). Disrupting both sites leads to synergistic ζ-globin reactivation.

This strategy was tested in several models, including a BHFS-like mouse model, HUDEP-2 cells, and primary human CD34+ cells from patients with HbH disease and BHFS. In primary patient-derived cells, ζ-globin reactivation restores α-like globin to approximately therapeutic levels (~50% after normalisation). Overall, the authors conclude that ζ-globin silencing is controlled by specific promoter elements and that editing these elements may provide a gene-editing approach for severe α-thalassaemia.

The study is novel, biologically elegant, and highly translational. It also suggests a broader therapeutic principle: reactivating developmentally silenced paralogues may be a useful strategy for other genetic diseases.

Minor comments:

1. The authors state that the proximity to α-globin enhancers may explain why a single Hba-alt gene produces about the same output as two endogenous α-globin genes. This point could be explained in more detail.

We thank the reviewer for this suggestion. This point has now been explained in more detail in the Main text, lines 117-120:

It is possible that the *Hba-alt* gene competes with endogenous α-globin genes for the enhancer activity, and its relative proximity to the enhancers places it in a more favourable position for frequent enhancer interactions.

2. Why was exon 3 chosen for replacement with mVenus?

In this ζ-globin-mVenus reporter mouse model, we replaced the last exon of the ζ-globin gene with mVenus, so that we could retain the structure of the ζ-globin gene and preserve all potential regulatory elements, including sequences within its promoter, introns and UTRs.

3. The BCL11A disruption had only a modest effect (~4% of total α-like globin). Was this because only sg33 was chosen? Would targeting a broader region, such as sg32–33, improve reactivation?

We agree that only one guide RNA may introduce small indels at BCL11A motifs and only cause a modest effect, so we generated clonal cell lines with complete deletion of the BCL11A motifs (Fig. 2d and Extended Data Fig. 2f). The results are consistent with our previous study (King et al., 2021), in which we have shown that BCL11A knockout alone has a modest effect on ζ-globin reactivation. We suspect that disrupting BCL11A alone, either by knocking it out or by mutating its binding site at the ζ-globin promoter, has a modest effect on ζ-globin reactivation.

4. Have the authors assessed potential off-target genomic events of editing the ζ-globin promoter?

We have not assessed the off-target effects of editing the ζ-globin promoter in this study. Because the current study was designed to establish biological proof-of-principle rather than develop a clinical product, comprehensive off-target assessment was considered beyond its scope. In the discussion, we addressed this as our plan, and our future work will focus on assessing the safety of these approaches in patient-derived HSPCs, including off-target effects and long-term engraftment.

5. Editing efficiency varied widely (25–90%). This should be discussed, especially given that HSC editing in clinical settings may be lower.

We thank the reviewer for this suggestion. We have added this point to the discussion, lines 392-395:

The efficiency of our current editing strategies varies in human primary HSPCs. Given that HSC editing in clinical settings may be lower, follow-up work will be needed to optimise editing strategies and delivery methods to achieve full clinical effectiveness.

6. The manuscript should also discuss lack of oral drugs for treating HbH disease until recently when mitapivat, a pyruvate kinase activator, that has recently been approved for treating both alpha and beta thalassaemia. Gene editing approach should be discussed in this context.

We thank the reviewer for this suggestion and agree that it is important to consider emerging therapeutic approaches for HbH disease, including mitapivat and other pharmacological interventions. A comprehensive comparison of genome editing with alternative therapeutic strategies would substantially broaden the scope of the discussion beyond the primary mechanistic focus of the current study. We have therefore limited our discussion to a brief contextual overview while focusing on the biological insights and proof-of-principle findings presented here.

We appreciate that pharmacological and gene-editing approaches may ultimately prove complementary, with pharmacological therapies targeting ineffective erythropoiesis and red cell metabolism, while gene editing offers the possibility of durable molecular correction.

Reviewer #3 (Remarks to the Author):

A. In this manuscript Liu et al. map cis-regulatory elements that silence embryonic alpha-globin gene (zeta) transcription with the aim of reactivating zeta-gene expression to ameliorate alpha-thalassemia eventually in a clinical setting. As a first step, they employ engineered mice to localize silencing elements to a 4.4 kb region containing the zeta-gene. Functional mapping with a Venus zeta-reporter mouse identified BCL11A and LRF binding motifs. Inactivation of both achieves substantial reactivation (~26%). To test the consequences of reactivation in a preclinical model, they proceed to use mice with a deletion that replicates the naturally occurring HbBarts allele (BHFS mice). The BHFS mice mimic the human phenotype. ZPE (zeta promoter edited) mice, in which both distal BCL11A and LRF motif are mutated, appear near normal at E17.5 (with expression of HbPortland II (zeta2beta2), suggesting "translational potential" in severe a-thalassemia. To pursue human relevance of their findings, they generate a HUDEP cell model, and examine consequences of mutation of the binding motifs in CD34 cells. In the human context, reactivation of zeta-globin appeared somewhat better than in mouse, which they interpret as reassuring for translation to patients. In essence, the manuscript has two aspects: 1) mapping of the sequences that silence zeta-globin gene expression in adult cells; and 2) preclinical studies for translation to severe alpha-thalassemia.

B. The studies in this manuscript are well executed. Principal novelty relates to the engineering and use of mouse alleles to reveal in vivo consequences of mutations. The mapping of the critical promoter regulatory sequences, culminating in identification of BCL11A and LRF motifs, is conventional. Prior studies (eg ref 23) pointed to those sequences. It is somewhat disappointing that the "missing" portion of silencing (ie that accounting for substantial residual expression in the context of combined BCL11A and LRF mutation) remains obscure. In terms of significance, the underlying hypothesis that zeta-globin reactivation might be a therapeutic approach to treatment of severe alpha-thalassemia rests primarily on the documented success of gamma-globin reactivation to provide functional cures of sickle cell disease and beta-thalassemia. Surprisingly, this logical foundation for the present studies is not mentioned in the text. This is a glaring omission. Prior genetic and family studies provided firm evidence that reactivation of gamma-globin expression would be of major therapeutic benefit. The same cannot be said regarding reactivation of zeta-globin expression (as noted in part F below). In the opinion of this reviewer, the authors are glib about the therapeutic benefits that might accrue from zeta-globin reactivation.

C. On the whole, the data and execution are excellent. The presentation is solid except for the issues that relate to translation of the findings (see below).

D. Statistical evaluation of results is generally solid with one exception: In the section "Identification of represive....mouse zeta-globin gene promoter", they describe the effect of combined BCL11A and LRF motif disruption as "synergistic", where individual mutation leads to 4 and 18% zeta-expression and the combined mutation 26% (which is not far from 22%, the sum of individual mutations).

We thank the reviewer for this point. We agree that this data should be interpreted cautiously. We have now tempered our interpretation accordingly, lines 161-163:

The double mutant cells show a higher (~26%) ζ-globin upregulation (Fig. 2d), suggesting a combined effect that is marginally greater than the sum of its parts.

E. The conclusions with respect to the consequences of mutation of the BCL11A and LRF motifs in the zeta-promoter are solid, although perhaps not very surprising.

F. Suggested improvements:

Specific points:

1. Fig 1a: the arrow from Hbq1b to Hba-x may be confusing. It is not explained what it is meant to depict (minor comment)

The grey arrow in Fig. 1a is from Hba-a1 (pink highlight) to Hba-x (blue highlight), indicating replacement of the Hba-x gene with a modified Hba-a1 gene. This is now explained in the figure legend, lines 695-697:

The blue highlight indicates the 4.4 kb region replaced by the modified α-globin (*Hba-alt*) gene in the DZIA model, the grey arrow from *Hba-a1* to *Hba-x* indicates the replacement.

2. The silencing elements are contained within the 4.4 kb region. The presumption is that they are in the promoter. In the experiments in which a Venus reporter is substituted for a portion of the zeta gene, can one exclude the possibility that some silencing sequences might be within the exon 3 deletion?

We thank the reviewer for this important point. We show that the silencing elements are contained within the 4.4 kb region, and our data are most consistent with predominant promoter-proximal control, while not excluding the possibility that other silencing sequences may also lie within this region. We agree that in the Venus knock-in reporter experiments, we cannot formally exclude the possibility that sequences within the deleted exon 3 contribute to silencing. However, the persistent repression in this reporter model suggests that exon 3 is unlikely to make a major contribution to ζ-globin silencing. We have expanded our discussion and proposed that other potential regulatory elements may contribute to ζ-globin silencing, lines 389-392:

In the future, these levels of ζ-globin expression may be further increased. While BCL11A and LRF appear to be the major repressors of ζ-globin expression in definitive erythroid cells, other elements with less effect may also exist [18]. In addition, there may be embryonic-specific activators that are missing in definitive erythropoiesis.

3. In the ZPE mouse model experiments, it is shown that augmented zeta expression improves the phenotype at E17.5. It is unexplained why liveborn mice (and perhaps older mice) were not studied. Given concerns regarding the in vivo functionality of Portland II, this experiment would seem important to provide.

We have revised the abstract to clarify that our conclusions are based on analysis of embryos close to birth at E17.5. At this stage, homozygous mutant embryos carrying

deletions of the LRF and BCL11A motifs appeared morphologically comparable to wild-type littermates and showed marked correction of erythroid abnormalities (Fig. 3g), associated with ζ-globin reactivation (Fig. 3e) and Hb Portland II ($\zeta_2\beta_2$) expression (Fig. 3f). These results suggest that Hb Portland II may be functional *in vivo*.

However, limitations on our current animal experimentation licence mean that we are not permitted to assess postnatal survival or long-term viability in case the level of zeta-globin reactivation in the mouse model is insufficient for a complete rescue. Allowing potentially severely affected animals to proceed to birth would raise animal welfare concerns on our current licence. Although we are currently unable to assess this, we do anticipate that the BHFS phenotype will be significantly ameliorated postnatally and will provide a potential treatment for this condition.

Proceeding with a plan to take this project from a proof-of-concept to therapeutic development, we are now applying for funding and setting up appropriate collaborations to demonstrate the efficacy of this approach postnatally. For example, animal study plans will include experiments with pre-natal and post-natal blood transfusions to allow 'treated' animals to be born and assess the efficacy and durability of the rescue postnatally and into adulthood. However, this work lies beyond the current study.

4. In lines 226-230, the authors note that the proximal BCL11A site overlaps the CCAAT box, precluding mutation of that site. However, perhaps the proximal site is part of the "missing" silencing? Would it be possible to engineer a mutation of the BCL11A site that preserves the CCAAT box? The authors should consider this possibility and perhaps perform an experiment to address this issue directly.

We agree that the proximal BCL11A-binding site (TGA<u>CCA</u>) may contribute to the residual silencing observed in our models. However, this site overlaps the <u>CCA</u>AT box, making it difficult to mutate without also altering an important core promoter element. Using currently available base editors, we were unable to design an editing strategy that would only engineer the BCL11A motif while preserving the CCAAT box. Any CRISPR/Cas9-HDR strategy is also challenging due to the highly conserved duplicated HBZP gene downstream, which can also be targeted, thereby creating large deletions. In the future, the availability of new base editors with more flexible PAM sites may make this possible. For the moment, we have acknowledged that the role of the proximal BCL11A site cannot be separated from the core promoter in the current study, lines 230-232:

…disrupting the proximal BCL11A binding motif does not reactivate ζ-globin expression (Fig. 4b), most probably because its role cannot be separated from the overlapping CCAAT box, which is essential for gene transcription [27, 28].

5. In lines 384/5, it is suggested that the inability to show disruption of binding of BCL11A and LRF is due to their "hit and run" action. This is a speculation. It is equally possible that the problem related to the closed nature of the target sites and inaccessibility (minor point).

Thank you for suggesting this point. We have added this to Discussion, lines 401-402:

It is also possible that BCL11A and LRF can bind their cognate motifs even within the context of 'close' chromatin, but this was not studied further here.

6. As noted above, in order to place this work in the proper context, the authors should cite parallel work on the reactivation of gamma-globin as a strategy to treat the beta-hemoglobin disorders.

Thank you for highlighting this. We have now reinforced this point and added references to the Discussion, lines 322-323 and lines 413-414:

Similar to recent developments in therapy for β-thalassaemia and sickle cell disease [41-44],…

…this work reinforces the emerging paradigm, illustrated by γ-globin reactivation in β-thalassaemia and sickle cell disease [41-44],…

41. Sankaran, V.G. and S.H. Orkin, *The switch from fetal to adult hemoglobin.* Cold Spring Harb Perspect Med, 2013. **3**(1): p. a011643.

42. Orkin, S.H., *The Fetal-to-Adult Hemoglobin Switch - Mechanism and Therapy.* N Engl J Med, 2025. **392**(21): p. 2135-2149.

43. Frangoul, H., et al., *CRISPR-Cas9 Gene Editing for Sickle Cell Disease and beta-Thalassemia.* N Engl J Med, 2021. **384**(3): p. 252-260.

44. Locatelli, F., et al., *Exagamglogene Autotemcel for Transfusion-Dependent beta-Thalassemia.* N Engl J Med, 2024. **390**(18): p. 1663-1676.

7. In this reviewer's opinion, the authors should be more critical in their assessment of the potential of zeta-globin reactivation to treat severe alpha-thalassemias. The strongest evidence in favor is ref 7 in which transgenic rescue of alpha-gene deleted mice was reported. In the published manuscript there are precious few details regarding the level of expression of the transgene and the hematology of the rescued mice. There are several reasons to suspect that Portland II may not substitute for HbA as well as implied in the current work. For example, as reported in Protein Science 16: 1641, 2007), the dimer strength of HbPortland II is far weaker than that of HbA or F, and the oxygen affinity is much higher. The difference in dimer strength may suggest that Portland II may be less stable. Since there are no data in the human context demonstrating that Portland II may substitute for HbA in vivo, I would suggest that the authors be more circumspect regarding the clinical application of the approach. The clinical potential of the approach will rest on not only the level at which zeta-globin is reactivated, but likely more importantly on the properties of Portland II and effects on cell lifespan and on oxygen delivery to tissues. These are largely unknown and the authors should acknowledge this.

We thank the reviewer for this thoughtful comment. We agree that the translational implications of ζ-globin reactivation should be discussed more cautiously. While the previous study (Russell and Liebhaber, 1998) and this study (Fig. 3e-g) support the proof-of-principle that ζ-globin expression would ameliorate α-globin deficiency *in vivo*, important questions remain regarding the extent to which Hb Portland II can functionally substitute for Hb A *in vivo*, particularly in the human context.

In contrast to the well-documented persistent expression of γ-globin in HPFH, there are rare reports of individuals with persistent ζ-globin expression. In our registry of BHFS survivors

(Songdej et al., 2017; Songdej DPhil thesis, 2015), we recorded one such case: a spontaneous survivor who was not diagnosed as BHFS until one year old, because their relatively mild phenotype was not consistent with a diagnosis of BHFS. This patient remarkably survived the first year of life on Hb Portland I and II, requiring only 5 blood transfusions.

As the reviewer points out, previous studies have shown that Hb Portland II is less stable than Hb A/F, and the oxygen affinity is higher (He et al., 2001; Maning et al., 2007). We have therefore revised the Discussion to more explicitly acknowledge these differences and emphasise that further analysis of the physiological properties of Hb Portland II in adult life will be required before clinical application. We have also acknowledged that the therapeutic threshold is likely determined not only by the level of ζ-globin expression but also by the biochemical and physiological properties of the resulting haemoglobin molecules.

Lines 303-310:

Despite weaker dimer interactions and higher oxygen affinity than Hb A ($\alpha_2\beta_2$) [36, 37], there is good evidence to suggest that the resulting haemoglobin molecule Hb Portland II ($\zeta_2\beta_2$) may be functional *in vivo* [7, 38] (Fig. 3e-g). Further analysis of the physiological properties of this haemoglobin in adult life will be required before clinical application. The therapeutic threshold is likely determined not only by the level of ζ-globin expression but also by the biochemical and physiological properties of the resulting haemoglobin molecules. Nevertheless, these results present a potentially viable and rational therapeutic strategy for the treatment of severe α-thalassaemia and pave the way for translational clinical trials.

36. He, Z. and J.E. Russell, *Expression, purification, and characterization of human hemoglobins Gower-1 (zeta(2)epsilon(2)), Gower-2 (alpha(2)epsilon(2)), and Portland-2 (zeta(2)beta(2)) assembled in complex transgenic-knockout mice.* Blood, 2001. **97**(4): p. 1099-105.

37. Manning, L.R., et al., *Human embryonic, fetal, and adult hemoglobins have different subunit interface strengths. Correlation with lifespan in the red cell.* Protein Sci, 2007. **16**(8): p. 1641-58.

38. Amid, A., et al., *Hemoglobin Bart's hydrops fetalis: charting the past and envisioning the future.* Blood, 2024. **144**(8): p. 822-833.

G. References: Citation of prior work on HbF reactivation should be added. Also,publications on the properties of Portland II (as above) should be included.

Thank you for highlighting this. We have now added the relevant references in the sections where we discussed HbF reactivation and properties of Portland II.
