# Supplementary material for: Reactivation of the embryonic ζ-globin gene ameliorates severe forms of α-thalassemia
Source: Nat Genet. 2026 Oct 1;58(10):2619–29. doi: 10.1038/s41588-026-02770-0 (PMC13649559; doi:10.1038/s41588-026-02770-0)
Supplement: Supplementary file 3 [file 41588_2026_2770_MOESM5_ESM.pdf]

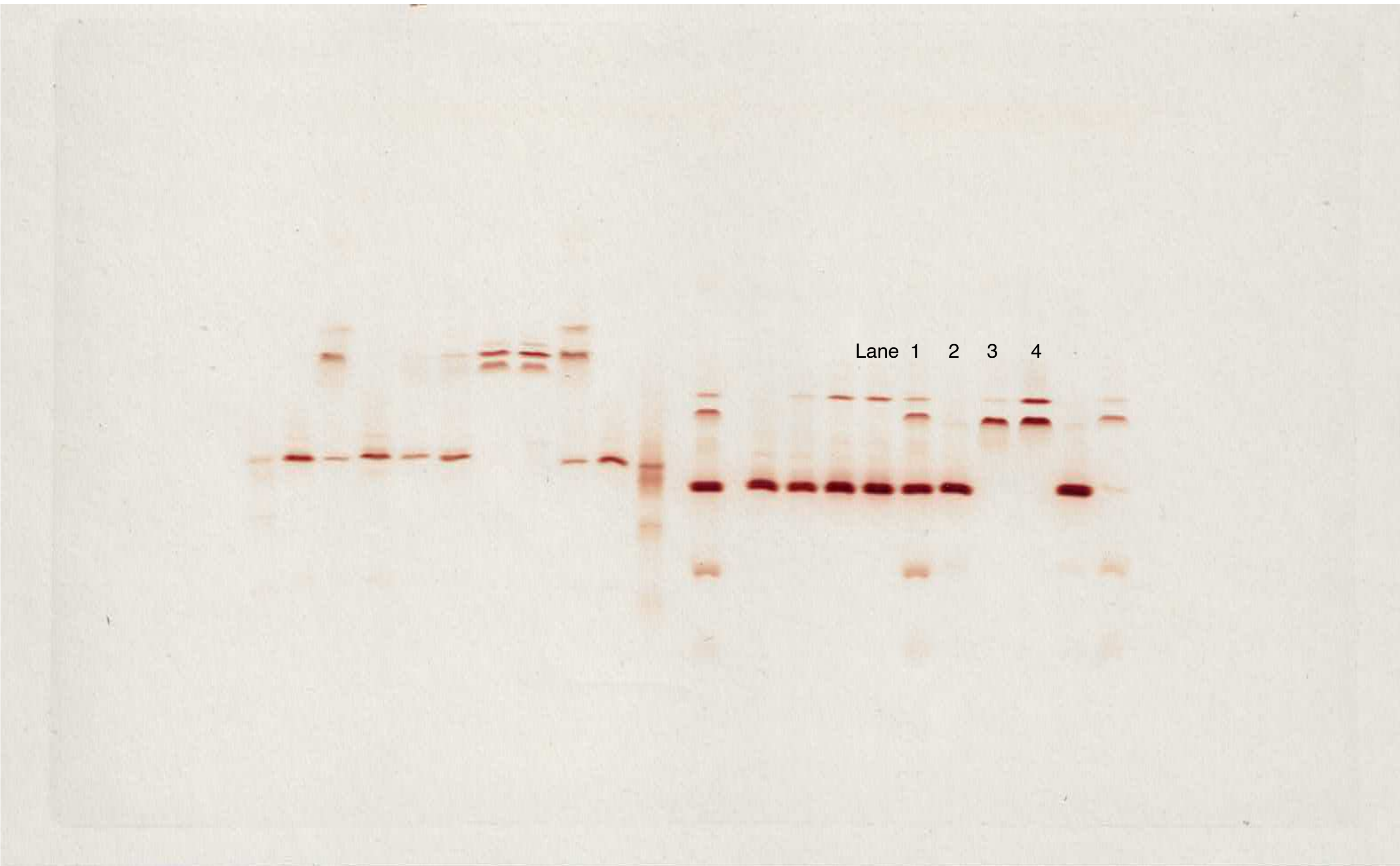
Lane 1 2 3 4
